# DPCDI: an artificial intelligent-derived indicator interpreting the diagnostic, stratification, and therapeutic implications of druggability programmed cell death in heart failure

**DOI:** 10.3389/fgene.2025.1753636

**Published:** 2026-01-15

**Authors:** Lili Zhang, Yihao Zhu, Yuan Fang, Yanping Yang, Yin Yu, Hanshi Wang, Xiyue Jiang, Xue Zhang, Dong Huang

**Affiliations:** 1 Department of Cardiology, Shanghai Sixth People’s Hospital Affiliated to Shanghai Jiao Tong University School of Medicine, Shanghai, China; 2 Center for Translational Medicine, Naval Medical University, Shanghai, China

**Keywords:** bioinformatics, cell death, heart failure, machine learning, multi-omics

## Abstract

Programmed cell death (PCD) pathways with druggable potential represent a promising but still underexplored frontier in heart failure (HF) research for diagnosis, prognosis, and therapy. To address this gap, we developed a Druggable Programmed Cell Death Index (DPCDI) through an integrative machine learning framework. An optimal combination of Lasso and Random Forest algorithms identified 15 pivotal genes (CALCOCO2, VPS13D, CLU, STAT3, OPTN, UBB, CXCL12, PPP1R15A, ATF4, IVNS1ABP, HMGB2, JAK2, EXOC7, ENO1, TPCN1) for DPCDI construction. Non-negative matrix factorization (NMF) analysis stratified HF patients into two distinct subtypes, with Subtype 2 exhibiting elevated apoptosis and mitophagy activity. Single-cell RNA sequencing revealed dynamic JAK2 and IVNS1ABP expression during cardiomyocyte state transitions, while CXCL12 showed stage-specific regulation in endothelial cells. Mendelian randomization analysis indicated that genetic predisposition to elevated JAK2 and STAT3 expression was associated with reduced HF risk, whereas CXCL12 overexpression increased susceptibility. Experimental validation in HF mouse models confirmed increased Cxcl12 and Jak2 expression and decreased Stat3 levels. Furthermore, knockout of Cxcl12, Jak2, and Stat3 induced HF phenotypes. Molecular docking identified pifithrin-α as a potent ligand for CXCL12 and strophanthidin for STAT3. Collectively, DPCDI provides a comprehensive framework for HF diagnosis, risk stratification, and targeted therapeutic development.

## 1 Introduction

Despite decades of laboratory research and clinical advances in heart failure (HF) treatment, global HF-related mortality and morbidity continue to rise (Heidenreich et al., 2022). According to the Global Burden of Disease Study 2017, an estimated 64.3 million people worldwide suffer from HF (GBD 2017 Disease and Injury Incidence and Prevalence Collaborators, 2018), imposing persistent medical and financial burdens. HF is a complex multifactorial clinical syndrome characterized by structural or functional impairment of ventricular filling and/or blood ejection (Bozkurt et al., 2021), leading to heart dysfunction. Its etiology involves diverse factors, notably ischemic heart disease, hypertension, and dilated cardiomyopathy (Savarese et al., 2023), making it impossible to attribute HF to a single cause. Given this high prevalence and etiological heterogeneity, efforts to improve HF management remain imperative. Consequently, novel biomarkers and therapeutic targets have garnered increasing interest for their potential to enhance preventive and therapeutic strategies (Shu et al., 2022).

Programmed cell death (PCD), an evolutionarily conserved process, plays a critical role in the pathogenesis of cardiovascular diseases including HF (Del Re et al., 2019; Zhou et al., 2021). Accumulating evidence indicates that dynamic alterations in druggable PCD pathways—such as apoptosis, necrosis, ferroptosis, and autophagy—drive HF progression (Zhou et al., 2021; Narula et al., 2006), which indicates an attractive therapeutic manipulation by targeting PCD. For example, administration of empagliflozin and sacubitril has been found to reduce apoptosis in rat models with HF symptoms, which demonstrates protective effects on cardiac functions (Jankowski et al., 2024). Additionally, different types of PCD signaling are interconnected at multiple levels in HF progression (Zhou et al., 2021), underscoring the need to focus on the interactions between various PCD patterns rather than an individual PCD. Over the past years, numerous efforts have been made to establish HF-predictive signatures using an individual PCD pattern (Jiang et al., 2022; Gu et al., 2023), and moderate diagnostic performances were observed in small-scale or different specimen-source cohorts. As such, leveraging multiple PCD forms to construct a signature based on single-source specimen cohorts may better represent the characteristic of HF than using a single PCD information. Also of note is that the biological mechanisms underlying most of these published signatures in HF were poorly understood, which greatly limits their future applications. A more profound comprehension of the mechanisms governing druggable PCD patterns in the context of HF, particularly their complex interactions, is crucial for advancing our current knowledge. However, to date, this in-depth understanding remains elusive.

To address these shortcomings, we aimed to comprehensively investigate the diagnosis, stratification, and therapeutic significance of four druggable PCD patterns (apoptosis, necrosis, ferroptosis, and autophagy), as well as elucidate the latent biological mechanisms in HF. Herein, we first designed an integrative machine learning (ML) framework to discover a Druggable Programmed Cell Death Indicator (DPCDI) on the basis of large expression profiling cohorts, which was used to identify and stratify patients with HF. Then, we implemented the single-cell RNA sequencing to expound the biological mechanisms underlying DPCDI in the context of the HF cell atlas. Using Mendelian randomization (MR) analysis, in-silicon drug prediction, and molecular docking, we also investigated the therapeutic applications of DPCDI in the treatment of HF. Altogether, we proposed a DPCDI with a potential implication in diagnosing, stratifying, and medicating patients with HF.

## 2 Methods

### 2.1 Data collection and processing of HF cohorts

Gene expression profiling of left ventricle (LV) from control and HF cases was downloaded from the GEO database (http://www.ncbi.nlm.nih.gov/geo). Herein, we collected a total of ten independent cohorts, including GSE141910 (Tan et al., 2020; Flam et al., 2022), GSE116250 (Yamaguchi et al., 2020; Sweet et al., 2018), GSE135055 (Hua et al., 2020), GSE16499 (Kong et al., 2010), GSE5406 (Hannenhalli et al., 2006), GSE57338 (Liu et al., 2015), GSE79962 (Matkovich et al., 2017), GSE42955, GSE52601, and GSE21610. Regarding the high throughput sequencing cohorts (GSE141910, GSE116250, GSE135055), the raw count value was converted to log_2_ (TPM+1) value. For the array cohorts (GSE16499, GSE5406, GSE57338, GSE79962, GSE42955, GSE52601, and GSE21610), the raw signal value was processed through quantile normalization and log2 transformation. We also enrolled GSE121893, a single-cell RNA sequencing data on LV and left atrium (LA) samples collected from four HF patients (Wang et al., 2020). Information on HF cohorts collected in this study can be found in Table 1. For detailed processing of GSE121893, see Methods-Expression patterns and dynamics of DPCDI at single-cell resolution.

**TABLE 1:** Information on HF cohorts collected in this study.

Accession	Type	Sample	Platform	Control	HF	Usage
GSE141910	RNA-seq	Left ventricle	GPL16791	166	200	Training
GSE116250	RNA-seq	Left ventricle	GPL16791	14	50	Testing
GSE135055	RNA-seq	Left ventricle	GPL16791	9	21	Testing
GSE16499	Array	Left ventricle	GPL5175	15	15	Testing
GSE5406	Array	Left ventricle	GPL96	16	194	Testing
GSE57338	Array	Left ventricle	GPL11532	136	177	Testing
GSE79962	Array	Left ventricle	GPL6244	11	20	External validation
GSE42955	Array	Left ventricle	GPL6244	24	5	External validation
GSE52601	Array	Left ventricle	GPL570	8	4	External validation
GSE21610	Array	Left ventricle	GPL10558	30	8	External validation
GSE121893	scRNA-seq	Left ventricle and atrium	GPL18573	2	4	scRNA-seq cohort

### 2.2 Development and external validation of DPCDI model

GSE141910, which contains the largest case numbers in our enrolled cohorts, was used as a training cohort for discovering a DPCDI model. And the other five cohorts (GSE116250, GSE135055, GSE16499, GSE5406, GSE57338, and GSE79962) were selected as testing cohorts for DPCDI validation. Before establishing DPCDI, we collected 1,066 druggability PCD-related genes (Supplementary Table S1), which encompass apoptosis, ferroptosis, autophagy, and necroptosis-related genes. Subsequently, we applied Self-Organizing Maps (SOM) to cluster the identified PCD-related genes based on their expression patterns in the GSE141910 dataset. The SOM grid dimensions were set to 10 × 10. Training was performed for 1,000 iterations, and the optimal number of clusters (k = 15) was determined by minimizing the within-cluster sum of squares (WCSS). This analysis yielded a distinct cluster of genes exhibiting high expression in HF. Here we designed an integrative ML framework, which was composed of 113 combinations derived from 12 ML algorithms (Zhu et al., 2024; Liu et al., 2022), to generate a DPCDI model for HF recognition. In this framework, least absolute shrinkage and selection operator (Lasso), random forest (RF), stepwise generalized linear model (Stepglm), and generalized linear model by likelihood-based boosting (glmBoost) (Zhu et al., 2024), were initially used to select features from SOM-identified PCD genes. Next, the other eight algorithms, including Ridge, elastic network (Enet), support vector machine (SVM), linear discriminant analysis (LDA), partial least squares regression for generalized linear models (plsRglm), gradient boosting machine (GBM), eXtreme Gradient Boosting (XGBoost), and NaiveBayes (Zhu et al., 2024), were implemented to establish prediction models on the selected features. AUC (Area Under the Receiver Operating Characteristic Curve) is an important indicator for evaluating the performance of binary classification models. It comprehensively reflects the model’s ability to distinguish between positive and negative samples by quantifying the area under the ROC curve. In the case of imbalanced sample classes, AUC can objectively reflect the model’s performance. In total, 113 combinations were generated after parameter tuning and ten-fold cross-validation, of which the best combination with the highest average AUC was regarded optimal. This optimal combination was termed DPCDI. We also assessed the AUC performance of DPCDI in four external validation cohorts (GSE79962, GSE42955, GSE52601, and GSE21610). To ensure productivity of the integrative ML framework and our DPCDI, the source code, scripts, processed datasets, and instructions have been archived in the Zenodo repository (https://doi.org/10.5281/zenodo.17918314).

### 2.3 Comparison between DPCDI and other published signatures

To compare the predictive power of DPCDI with other signatures, we gathered 51 mRNA signatures for HF prediction that were published in the last 5 years, which was summarized in Supplementary Table S2. These mRNA signatures were established by diverse algorithms, such as RF, Lasso, Boruta, and SVM (Guo and Xu, 2023; Bian et al., 2022). Additionally, these signatures were derived from different biological processes, such as cellular senescence (Guo and Xu, 2023), immune microenvironment (Wang et al., 2024), and N7-methylguanosine modification (Ma et al., 2023). Model 50 (NPPA and NPPB) and model 51 (TNNT2 and TNNI3) integrate the genes corresponding to the two categories of traditional cardiac markers, respectively. For equitable comparability, we filtered out the signatures with more than 30% of genes not matched in cohorts for DPCDI training and testing. We calculated the AUC performance for each signature in all cohorts.

### 2.4 Construction of DPCDI-derived subtypes

We initially applied Rank-In (Tang et al., 2021), a well-established approach to combine the high throughput sequencing and array data, to integrate all cohorts into a meta-cohort for HF subtype discovery. We extracted 657 HF cases from the meta-cohort for subtype analysis. According to the expression profiling of DPCDI genes from these HF cases, the Non-negative Matrix Factorization (NMF) technique (Gaujoux and Seoighe, 2010) was performed to partition the HF patients into different subtypes. To ensure a stable identification, we used the brunet approach, with a setting of 100 iterations, to execute the NMF process. The cophenetic coefficient was used to determine the optimal rank for clustering (Gaujoux and Seoighe, 2010), and the optimal rank (=2) was selected to construct two subtypes. Furthermore, the principal components analysis (PCA) plot was used to assess the dispersion of the identified two subtypes. We also investigated the expressions of DPCDI genes and HF markers between the two subtypes.

### 2.5 Biological mechanisms and enrichment analysis of DPCDI

To understand the detailed biological peculiarities of DPCDI, we performed Gene Ontology (GO)-Biological Process (BP) and Kyoto Encyclopedia of Genes and Genomes (KEGG) enrichment analyses using the clusterProfiler package (Yu et al., 2012). The GO-BP and KEGG enrichment terms with P value <0.05 and PCD correlation were retained. Subsequently, we used the clusterProfiler package to implement Gene Set Enrichment Analysis (GSEA), to evaluate the enrichment distribution of the DPCDI-related terms across the two HF subtypes. The single-sample Gene Set Enrichment Analysis (ssGSEA) was also conducted using the GSVA package (Hänzelmann et al., 2013), to calculate the enrichment scores of these DPCDI-related terms. To delve into the biological peculiarities underlying DPCDI in HF progression and two HF subtypes, the KEGG database (https://www.genome.jp/kegg/) was acquired to depict the cross-talk between DPCDI genes and their enrichment terms.

### 2.6 Expression patterns and dynamics of DPCDI at single-cell resolution

We used GSE121893, which contains single-cell RNA sequencing information on LA and LV samples from four HF patients (Wang et al., 2020). Consistent with our previous analysis of LV cohorts, we retained LV samples for subsequent exploration. Herein, the single-cell RNA sequencing analysis on GSE121893 was accomplished by the Seurat package (Hao et al., 2021). Initially, we eliminated the poor-quality cells with gene count (less than 200 or larger than 5, 000) and mitochondrial proportion larger than 20%, and a total of 1,668 cells were retained. Next, we carried out a log transformation to normalize gene expression and identified the top 2,000 highly variable genes for downstream reduction. We then conducted a linear transformation to scale the gene expression, which assigns each gene the same weight. According to the scaling data, PCA was used based on the top 2,000 highly variable genes. Batch correction was performed using the harmony package with the following parameter settings (reduction = “pca”, dims = 1:15, max.iter = 20). The optimal number of PC (=15) was determined and subjected to Uniform Manifold Approximation and Projection (UMAP) clustering. Afterward, we obtained five different clusters based on the parameters setting (dimension = 1:15, resolution = 0.5). The identities of clusters were annotated using the well-known gene markers of heart cells (Wang et al., 2020; Hu et al., 2023). Thus, 5 cell populations were annotated: (1) Cardiomyocyte (CM), as reflected with higher expressions and proportions of MYL2, MYH7, FHL2, TTN, TNNT1, and TNNT2; (2) Endothelial cell (EC), as reflected with higher expressions and proportions of VWF, PECAM1, CDH5, and IFI27; (3) Fibroblast (FB), as reflected with higher expressions and proportions of ACTA2, CALD1, and MYH11; (4) Smooth muscle cell (SMC), as reflected with higher expressions and proportions of DCN, GSN, C7, LUM, FBLN1, and COL1A2; (5) Macrophage (MP), as reflected with higher expressions and proportions of PTPRC, CD163, CCL4, CXCL8, and LAPTM5. We next explored the expression patterns of DPCDI genes in these 5 cell populations. Additionally, we used the CellChat package (Jin et al., 2021) to quantitatively infer and analyze cell-cell communication, thereby understanding the intricate cell atlas in the context of HF.

Regarding the DPCDI genes-enriched cell population (CM and EC), we subsequently annotated their sub-populations. Next, we investigated the expression patterns of DPCDI genes in these defined sub-populations of CM and EC. Using the monocle3 package (Cao et al., 2019), we proposed a pseudo-time analysis to infer the transition trajectory across the CM or EC sub-populations, so as to expound the dynamics of DPCDI genes. The inferred trajectory was then projected to a UMAP plot for visualization. To gain insight into a comprehensive molecular program underlying the transition, we analyzed the Differential Expressed Genes (DEGs) that significantly changed among the pseudo-time under the threshold of Moran’s I > 0.05 and Q-value <0.05. The ClusterGVis package (Zhang, 2022) was used to divide these DEGs into distinct patterns using K-means clustering. GO and KEGG enrichment analyses were implemented for each pattern using the clusterProfiler package (Yu et al., 2012), and the enriched terms with P < 0.05 were regarded as significant.

### 2.7 Mendelian randomization of the causal-effect between DPCDI expression and HF risk

Two-sample Mendelian randomization (2SMR) analysis (Hemani et al., 2018) was used to assess the causal association between the genetic predisposition of three DPCDI genes (CXCL12, JAK2, STAT3) and HF. This data was retrieved from the IEU Open GWAS (https://gwas.mrcieu.ac.uk/) and deCODE (https://www.decode.com/summarydata/) (Ferkingstad et al., 2021; Elsworth et al., 2020). The population source of the database is Europe, with samples derived from peripheral blood. The exposure IDs were summarized as follows: JAK2-eQTL (eqtl-a-ENSG00000096968 in IEU Open GWAS); STAT3-eQTL (eqtl-a-ENSG00000168610 in IEU Open GWAS); CXCL12-pQTL (3516_60_CXCL12_SDF in deCODE); JAK2-pQTL (11816_84_JAK2_JAK2 in deCODE); STAT3-pQTL (10346_5_STAT3_STAT3 in deCODE); HF (ebi-a-GCST009541 in IEU Open GWAS). Following the 2SMR assumption, the Single Nucleotide Polymorphisms (SNPs) closely related to DPCID genes (P-value <5 × 10–8) but not with HF (P-value >0.05) were obtained. To avoid linkage disequilibrium (LD), we excluded the SNPs with LD-R2 greater than 0.01 within a cropping range of 5,000 Kb. Eventually, the assumption-compliant SNPs, with an F-statistic greater than 100, were retained as strong Instrumental Variables (IVs) for MR analysis. The inverse variance weighted (IVW) method, with the highest statistical power in 2SMR, was used to expound the causality between DPCDI genes and HF risk. Additionally, the Bayesian weighted Mendelian randomization (BWMR) (Zhao et al., 2020) was used to validate the results of 2SMR. To further explore the biological significance of these three DPCDI genes, the Mouse Genome Informatics (MGI) database (https://www.informatics.jax.org/) was accessed to show the cardiovascular phenotypes of CXCL12-, JAK2-, and STAT3-knockout mouses.

### 2.8 Heart failure mouse model and echocardiographic assessment

The animal experiments were approved by the Institutional Animal Ethics Committee of Shanghai Jiao Tong University (Approval number: 2025-0821), and complied with NIH guidelines. Male and female C57BL/6 mice (8 weeks old) were housed under standardized conditions (24 °C ± 2 °C, 40% ± 5% humidity, 12-h light/dark cycle). HF was induced by Transverse Aortic Constriction (TAC). Sham-operated mice underwent identical procedures without aortic ligation. Ejection fraction (EF) and fractional shortening (FS) were assessed by echocardiography 24 h after surgery. Heart tissues were harvested at 1 month post-surgery for analysis. Biometric data (EF, FS, body weight, and heart weight) are presented in Supplementary Table S3.

### 2.9 Hematoxylin and eosin (H&E) and masson staining

Heart tissues were fixed, paraffin-embedded, and cut into sections. Tissue morphology and fibrosis were assessed by hematoxylin and eosin (H&E) and Masson’s trichrome staining according to standard protocols. The infarct size and fibrosis area were quantified using ImageJ software.

### 2.10 RNA isolation and quantitative real-time PCR (qPCR) analysis

Total RNA from mouse heart tissue was isolated via an EZ-press RNA Purification Kit (EZBioscience). Reverse transcription to cDNA was performed with an RT Kit (EZBioscience). Quantitative PCR was performed via a Roche480 LightCycler® 96 real-time PCR system with 2×SYBR Green qPCR Master Mix (EZBioscience). The primer sequences are listed in Supplementary Table S4. An unpaired t-test was applied to assess the differences in gene expression between the control and HF groups. Pearson’s correlation analysis was used to examine the relationship between gene expression levels and echocardiographic indicators (EF and FS).

### 2.11 Drug prediction and molecular docking of DPCDI

The L1000 fireworks display (L1000FWD) database (https://maayanlab.cloud/l1000fwd/) (Wang et al., 2018) was queried to identify the small-molecule drugs targeting CXCL12 and STAT3, which displayed an opposite relationship with the expressions of CXCL12 and STAT3 in HF. According to the information on toxicity and availability of predicted drugs, we selected pifithrin and strophanthidin as the candidate drugs with potential utility in treating HF. Next, we conducted molecular docking to validate the reliability of the drug-target interaction based on the binding affinity and pattern (Yu et al., 2024a). We first downloaded the three-dimensional structures of pifithrin (PubChem CID: 4817) and strophanthidin (PubChem CID: 6185) from the PubChem website (https://pubchem.ncbi.nlm.nih.gov/), which was subjected to energy minizine using the ChemBio3 software. Next, we gained the three-dimensional structures of CXCL12 (ID: 3HP3) and STAT3 (ID: 6NJS) from the Protein Data Bank (https://www.rcsb.org/). The source of these protein structures was all X-ray crystal detection and derived from *Homo sapiens*. PyMOL software was used to preprocess the protein structures, including the removal of solvents, ligands, and hydrogens. To determine the active pockets of CXCL12 and STAT3 for docking drugs, we employed the AutoDock Vina software to perform polar hydrogenation and optimize the docking site. AutoDock Vina software was then utilized to dock the target proteins and drugs, including the interactions of CXCL12-pifithrin, CXCL12-strophanthidin, STAT3-pifithrin, and STAT3-strophanthidin. Subsequently, the affinity was computed to appraise the binding efficiency for each drug-target interaction, with an affinity lower than 5.0 kcal/mol indicating a superior bind efficiency. We visualized the binding pattern of each drug-target interaction using the PyMOL software.

## 3 Results

### 3.1 DPCDI derived from an integrative ML framework in multi-cohorts enables an accurate HF diagnosis

**FIGURE 1:**
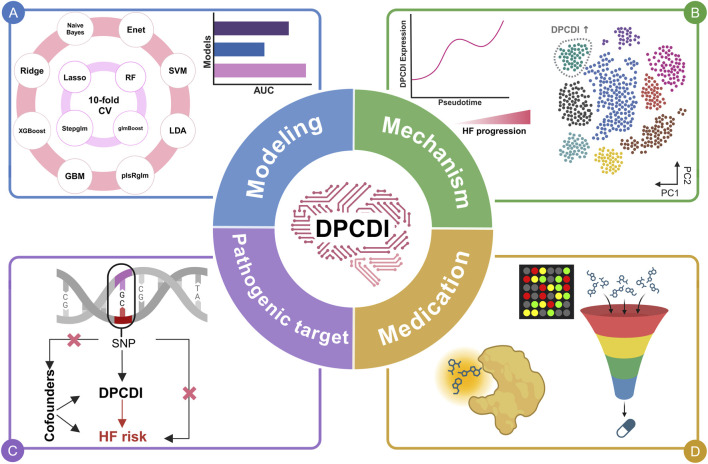
Overview of this study. **(A)** DPCDI modeling. **(B)** Biological mechanisms underlying DPCDI. **(C)** Pathogenic potential of DPCDI. **(D)** Therapeutic applications of DPCDI.

**FIGURE 2:**
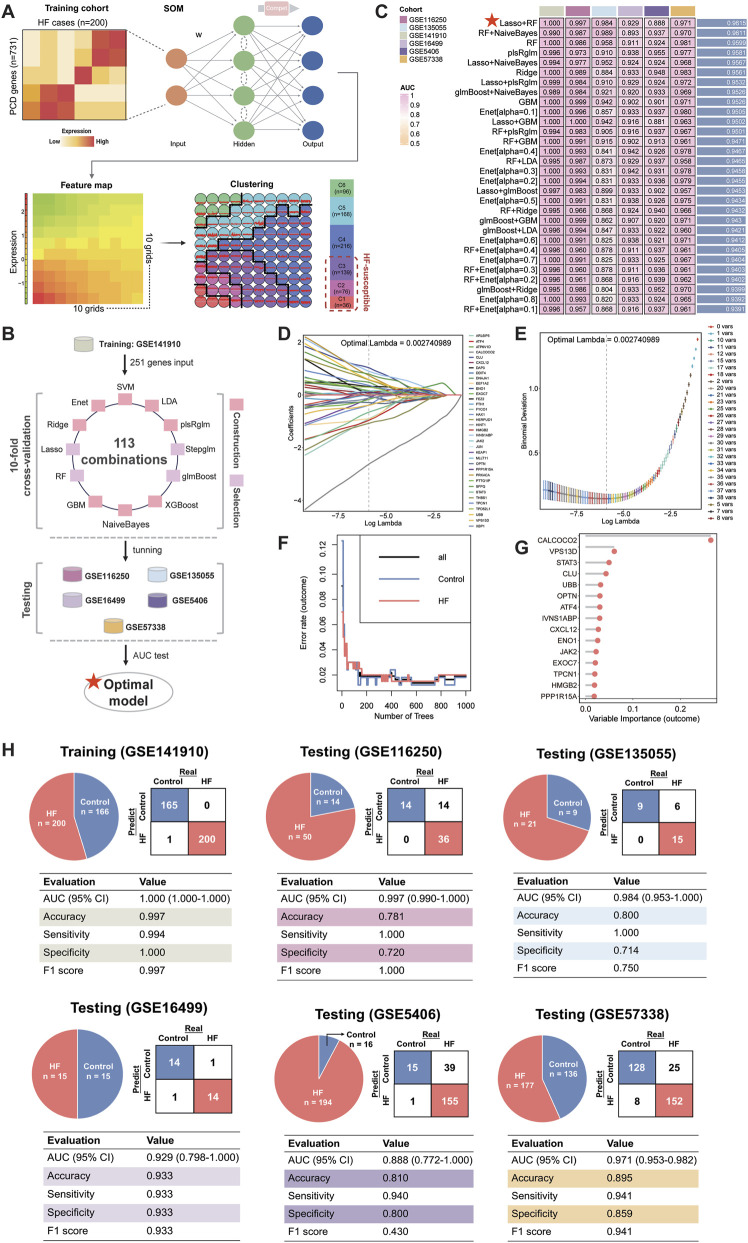
Establishment of an HF-predictive DPCDI using an integrative ML framework. **(A)** SOM identification of three sets of HF-susceptible genes in the training cohort. **(B)** Design of the integrative ML framework. **(C)** The AUC performance of 113 ML combinations for distinguishing HF from control across the training and five independent testing cohorts was sorted, and top 30 combinations was visualized as a heatmap. The best combination (Lasso and RF) with the highest average AUC was marked with a red star. **(D)** Lasso coefficients of 176 DPCD-related genes under optimal λ construction. A total of 36 genes with non-zero coefficients were retained (labeling on the right panel). **(E)** The minimum deviance was reached under the 36 Lasso-selected genes. **(F)** Changes in error rates using different tree numbers in RF. **(G)** Feature importances of 15 DPCD-related genes screened from 36 Las-so-selected genes via RF. **(H)** Confusion matrix and diagnostic assessment of DPCDI within the training and five independent testing cohorts. Each cohort was displayed respectively.

A flowchart outlining our study is delineated in Figure 1. SOM was initially performed in the training cohort, revealing a total of 251 DPCD-related genes that were actively expressed in HF (Figure 2A). DPCD-related genes assigned in each SOM-identified cluster were summarized in Supplementary Table S5. Subsequently, the expression profile of 251 DPCD-related genes from the training cohort was subjected to an integrative ML framework to develop a DPCDI, which was depicted in Figure 2B. In this computational framework, we established 113 kinds of algorithmic combinations under the ten-fold cross-validation and calculated the AUC score of each combination in all cohorts to assess their predictive performances. As shown in these 113 combinations, we noted that the best-performing combination, consisting of Lasso and RF algorithms, achieved the highest mean AUC (0.9815) across the training and five independent testing cohorts (Figure 2C). The AUC performances of all combinations can be found in Supplementary Data 2. Lasso identified the most valuable 36 DPCD-related genes with non-zero coefficients via the optimal lambda (Figure 2D), with a regression binominal deviation reaching minimum (Figure 2E). These 36 DPCD genes were then subjected to an RF algorithm using 1,000 trees (Figure 2F), and a final set of 15 genes to build DPCDI was identified, including CALCOCO2, VPS13D, CLU, STAT3, OPTN, UBB, CXCL12, PPP1R15A, ATF4, IVNS1ABP, HMGB2, JAK2, EXOC7, ENO1, and TPCN1 (Figure 2G). Moreover, we displayed the case distribution and confusion matrix of DPCDI in each cohort (Figure 2H). Four indicators for evaluating DPCDI prediction (accuracy, sensitivity, specificity, and F1 score) were then calculated in each cohort. Remarkably, we observed a relatively higher sensitivity of DPCDI, above 0.9 in all cohorts, suggesting a potential capability of DPCDI to accurately recognize HF. We also verified the robust predictive power of DPCDI in four external cohorts (GSE79962, GSE21610, GSE42955, and GSE52601), as demonstrated in Supplementary Figure S1. Overall, we provided a 15 gene signature, termed DPCDI fitted by Lasso and RF, as an attractive panel to predict HF occurrence.

### 3.2 Comparison of diagnostic performance of DPCDI and published HF-predictive signatures

**FIGURE 3:**
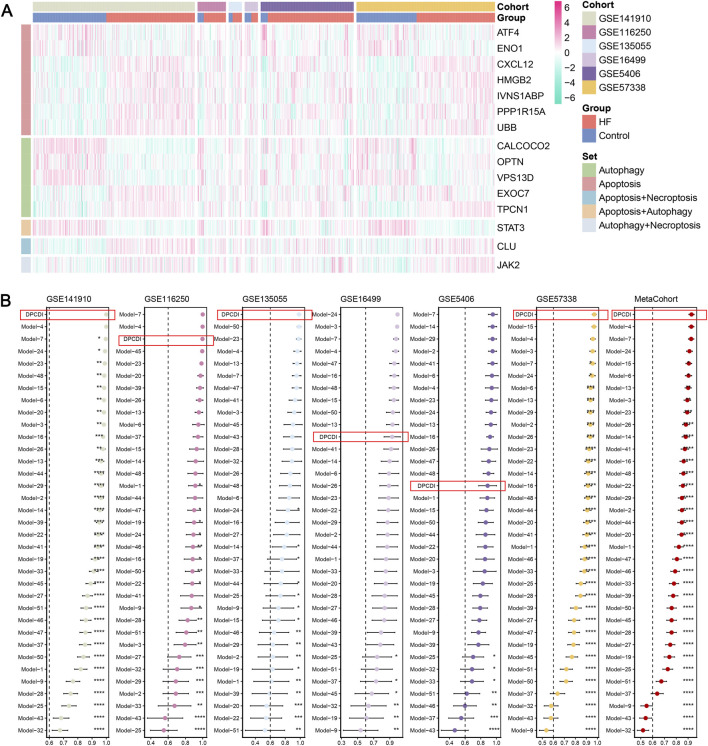
Superior predictive capability of DPCDI. **(A)** Heatmap displaying the expressions of DPCDI genes between control and HF cases within the training and five independent testing cohorts. **(B)** Comparison of diagnostic performance of DPCDI and other 50 published signatures. The AUC performance of DPCDI in each cohort was marked in a red bracket respectively. *, *P* < 0.05; **, *P* < 0.01; ***, *P* < 0.001; ****, *P* < 0.001.

We analyzed the expression landscape of altered DPCDI genes in each cohort, as displayed in Figure 3A. Expression changes in these genes were consistent in all cohorts, in which ATF4, ENO1, CALCOCO2, OPTN, VPS13D, and STAT3 were downregulated in HF. The log_2_fold change (log_2_FC), p-value and False Discovery Rate (FDR) of these genes between control and HF cases across cohorts were summarized in Supplementary Table S6. Whereas, CXCL12, HMGB2, IVNS1ABP, PPP1R15A, UBB, EXOC7, TPCN1, CLU, and JAK2 showed up-regulations in HF. These DPCDI genes were largely related to apoptosis or autophagy. Interestingly, these genes are expressed differently in HF, suggesting their intricate regulation in apoptosis and autophagy. To enable an equitable comparison of our DPCDI and other HF-predictive signatures, we systemically incorporated 51 gene signatures published within the past 5 years (see Methods). We filtered out signatures with larger than 30% missing genes in our enrolled cohorts, and a final set of 35 signatures was retained for comparison. These signatures were different functional gene panels, such as cellular senescence, immune microenvironment, and N7-methylguanosine. Notably, our DPCDI demonstrated better AUC performance than almost most signatures in the training (GSE141910), three testing (GSE116250, GSE135055, GSE57388), and meta-cohort (Figure 3B). Regarding the other two testing cohorts (GSE16499 and GSE5406), the AUC performance of DPCDI remained relatively robust (larger than 0.85) even though it was not the top-ranked model. Also worthy of note is that some signatures possessed better performance in their discovery cohort but were weak in other cohorts, which may arise from an over-fitting troublesome. For instance, the AUC of model three ranked second in the GSE16499 cohort, but relatively weakened within GSE116250 (ranked 27/35) and GSE5406 (ranked 21/35). In sum, our DPCDI showed a superior predictive performance than almost most published signatures, suggesting its extrapolation possibility with better generalization.

### 3.3 Partitioning of DPCDI-derived molecular subtypes for HF

**FIGURE 4:**
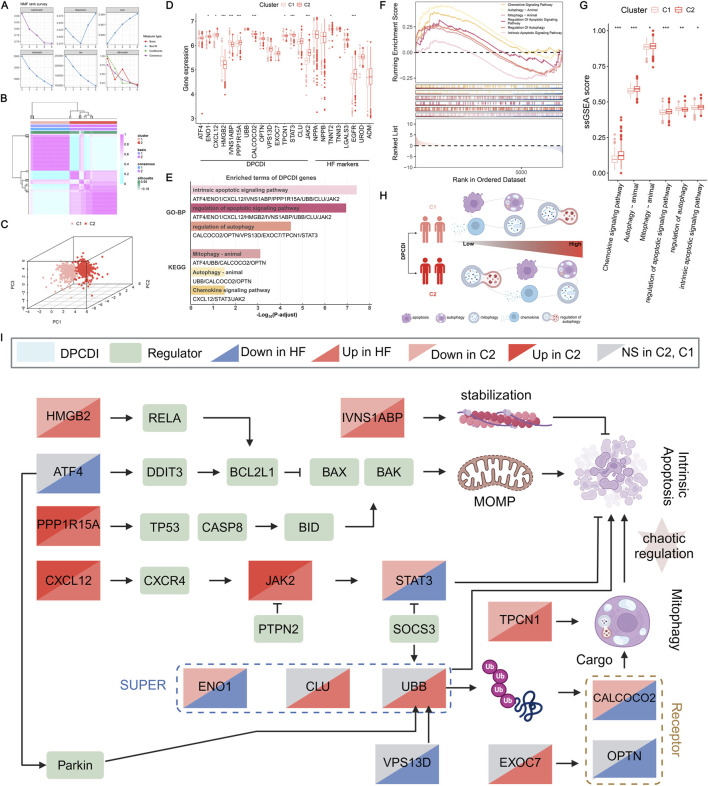
Construction of a DPCDI-derived HF subtype and biological peculiarities of DPCDI. **(A)** The distribution of cophenetic, residuals, RSS, and silhouette through ranks from 2 to 10. **(B)** Consensus map of NMF clustering when rank equals 2. **(C)** The three-dimensional PCA plot shows the distinct distribution of two clusters. **(D)** The expressions of DPCDI genes and HF biomarkers between control and HF. **(E)** Enriched GO and KEGG terms of DPCDI genes. **(F)** GSEA enrichment plot of DPCDI-related terms. **(G)** ssGSEA enrichment plot of DPCDI-related terms. **(H)** Schematic chart illustrating the differences in enrichment of DPCDI-related terms between HF and control cases. **(I)** Overview depicting the cross-talk between DPCDI genes and regulators.

To identify the HF molecular subtypes with different degrees, we performed NMF clustering to explore the molecular features of these DPCDI genes in HF. Based on the NMF metrics of cophenetic, residuals, RSS, and silhouette (Figure 4A), the suitable cluster number (rank = 2) was determined to subtyping. Accordingly, the HF cases merged in meta-cohort (N = 657) were successfully classified into two distinct clusters (Figure 4B), and this apparent dispersion was also demonstrated in the PCA plot (Figure 4C). As shown in Figure 4D, we also measured the expression landscape of DPCDI genes and HF markers among the C1 (N = 297) and C2 clusters (N = 360). Among the DPCDI genes, the significant downregulation of HMGB2, IVNS1ABP, CALCOCO2, and STAT3 (P-value <0.001) were observed in C2, while PPP1R15A and JAK2 showed the elevated pattern (P-value <0.001). We also found that TNINT2 and EGFR were significantly upregulated in C2.

To delve into the different molecular characteristics of DPCDI between these two clusters, we initially implemented the GO and KEGG enrichment analyses on the DPCDI genes. Detailed information on these analyses can be found in Supplementary Table S7. We showed that the intrinsic apoptotic signaling pathway, regulation of apoptotic signaling pathway, and regulation of autophagy represent the GO functional terms (Figure 4E). Additionally, we found that mitophagy, autophagy, and chemokine signaling pathway were the significant KEGG pathway terms, of which mitophagy integrates ATF4, UBB, CALCOCO2, and OPTN, and chemokine signaling pathway links CXCL12, JAK2, and STAT3. GSEA was then performed to measure the enrichment distribution of these functional and pathway terms between the C1 and C2 clusters. As shown in Figure 4F, we noticed that the intrinsic apoptotic signaling pathway, regulation of apoptotic signaling pathway, mitophagy, autophagy, and chemokine signaling pathway were highly enriched in C2 (NES >1; Supplementary Table S8), but regulation of autophagy was lowly enrichment (NES <1; Supplementary Table S8). Using the ssGSEA approach, we also observed the same tendencies in these DPCDI-enrichment terms between C1 and C2 (Figure 4G). Consequently, we observed that patients in C2 exhibited enhanced activity in apoptosis and autophagy-related pathways, specifically intrinsic apoptosis and mitophagy, while demonstrating reduced regulation of autophagy initiation (Figure 4H). We next investigated the interplays between the DPCDI genes in these biological processes, as depicted in Supplementary Figure S2. A pathway overview of crosstalk between the DPCDI genes and their regulators was summarized in Figure 4I. Interestingly, we noted that CALCOCO2 and TPCN1, two important regulators of autophagy cargo recognition, were decreased in C2, which partly explained the inhibited autophagy regulation in C2. More intriguingly, we found that the downstream STAT3 in the chemokine signaling pathway, which is supposed to be activated by CXCL12 and JAK2, was significantly downregulated in C2 patients. This data suggests that the C2 subtype exhibits more active apoptotic and autophagic processes, but the regulation of autophagy is impeded. Notably, the C1 and C2 subtypes were identified using transcriptomic data, in the absence of correlated clinical parameters such as baseline cardiac function or comorbidity profiles. Consequently, this observed differences between subtypes must be interpreted cautiously and require validation through future studies.

### 3.4 Expression patterns of DPCDI in HF cell atlas at single-cell resolution

**FIGURE 5:**
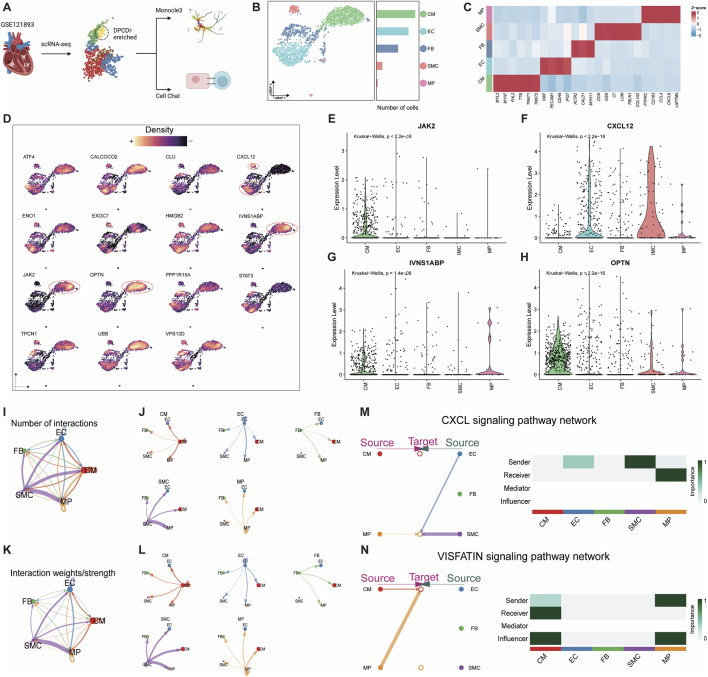
Exploration of DPCDI expression in HF patients at single-cell resolution. **(A)** Schematic illustration of this flow. **(B)** UMAP visualization and numbers of five annotated cell populations isolated from LV of HF patients. **(C)** Heatmap displaying marker genes for annotating each cell population. **(D)** Expression of DPCDI genes among the cell populations. Expression distribution of IVNS1ABP **(E)**, JAK2 **(F)**, OPTN **(G)**, and CXCL12 **(H)** among the cell populations. **(I)** The number of interactions of the five populations. **(J)** The number of interactions of each cell population. **(K)** The interaction weights/strength of the 5 cell populations. **(L)** The interaction weights/strength of each cell population. The line color and width represent cell type and interaction pairs, respectively. **(M)** Identification of CXCL signaling pathway network between EC, SMC, and MP. Left and right portions show the interactions and signaling roles in CXCL signaling pathway, respectively. **(N)** Identification of VISFATIN signaling pathway network between MP and CM. The left and right portions show the interactions and signaling roles in the VISFATIN signaling pathway, respectively.

We next questioned whether the expression patterns of DPCDI genes differ by the cellular diversity of LV. We applied scRNA-seq analysis of individual cells isolated from LV tissues of four HF patients. The workflow was shown in Figure 5A. A total of 1,668 high-quality cells were gained after data trimming and filtering (Supplementary Figure S3). Then, we performed unsupervised clustering to partition 1,668 cells that were identified as five distinct clusters using UMAP. Five main cell populations, including cardiomyocyte (CM), endothelial cell (EC), fibroblast (FB), smooth muscle cell (SMC), and macrophage (MP), were subsequently annotated based on their respective markers (Figures 5B,C). Next, we visualized the global expression of DPCDI genes among these 5 cell populations (Figure 5D). Worthy of noting is that the IVNS1ABP (Figure 5E), JAK2 (Figure 5F), and OPTN (Figure 5G) were highly expressed in the CM population. Nonetheless, STAT3 was almost absent in CM compared to other populations, suggesting its dysfunction despite JAK2 being actively expressed. Moreover, we noticed that CXCL12 expression was enriched in EC and SMC populations (Figure 5H). Interestingly, the CM population was only enriched for JAK2 activation but lacked CXCL12 and STAT3, yet the close relationship of the CXCL12-JAK2-STAT3 axis in chemokine signaling was reported. Thus, it is believed that CM and NCM populations interact dynamically in the context of HF. Given this specific DPCDI gene expression in CM and EC, we selected these cell types for further investigation.

CellChat, based on the information on ligands, receptors, and cofactors from scRNA-seq data, was implemented to quantitatively infer and analyze intercellular communication. The cell-to-cell interaction among CM, EC, FB, SMC, and MP was respectively visualized as circular plots by interaction number (Figure 5I) and interaction strength (Figure 5J). The cell-to-cell interaction for each population was respectively exhibited by interaction number (Figure 5K) and interaction strength (Figure 5L). Using cell centrality analysis, we next extrapolated the roles of cell populations in signaling pathways. Figure 5M shows the inferred intercellular communication network for CXCL12 signaling. Of note, EC and SMC are dominant senders, and MP is the receiver of CXCL12 signaling. Specifically in the most important contribution for ligand-receptor, EC and SMC highly expressed CXCL12 ligand, which acts as a sender to active CXCR4 receptor in MP (Supplementary Figure S4A). We also showcased an inferred intercellular communication network for VISFATIN signaling (Figure 5N), consisting of MP as a sender and CM as a receiver. Interestingly, both MPs and CMs are predicted to be influencers, suggesting their roles as interactive controls in VISFATIN signaling. Given that MPs are activated via CXCL signaling, we speculated that these pro-inflammatory MPs show the activity of the NAMPT ligand and subsequently activate the INSR ligand of CMs (Supplementary Figure S4B), which may drive the regulation of JAK2 in CMs.

### 3.5 Dynamics of DPCDI was observed during the transition of sub-populations of CM and EC

**FIGURE 6:**
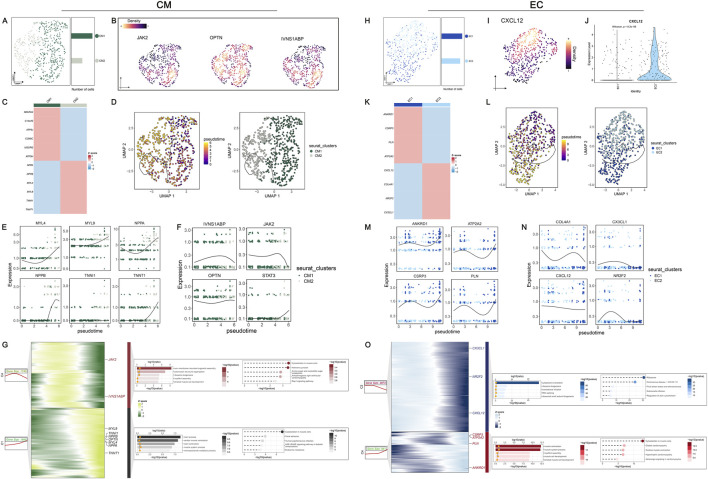
In-depth exploration of DPCDI dynamics in sub-populations of CM and EC during HF progression. **(A)** UMAP visualization and numbers of CM population. **(B)** Global expression of JAK2, OPTN, and IVNS1ABP among the CM population. **(C)** Heatmap displaying marker genes for CM1 and CM2 sub-populations. **(D)** Inference of pseudo-time trajectories of CM population. The left and right portions represent the branch trajectories with cells colored by pseudo-time and branch trajectories with cells colored by sub-population, respectively. **(E)** Expression dynamic of marker genes (MYL4, MYL9, NPPA, NPPB, TNNI1, TNNT1) across the pseudo-time. **(F)** Expression dynamic of IVNS1ABP, JAK2, OPTN, and STAT3 across the pseudo-time. **(G)** Gene dynamic analysis of CM population transition. The left part was a heatmap displaying the expression dynamics of DEGs with distinct patterns along the pseudo-time. The right part was GO and KEGG enrichment terms of each cluster. **(H)** UMAP visualization and numbers of EC population. **(I)** Global expression of CXCL12 among the EC population. **(J)** Expression distribution of CXCL12 between EC1 and EC2 sub-populations. **(K)** Heatmap displaying marker genes for EC1 and EC2 sub-populations. **(L)** Inference of pseudo-time trajectories of EC population. The left and right portions represent the branch trajectories with cells colored by pseudo-time and branch trajectories with cells colored by sub-population, respectively. **(M)** Expression dynamic of EC1-marker genes (ANKRD1, ATP2A2, CSRP3, PLN) across the pseudo-time. **(N)** Expression dynamic of EC2-marker genes (COL14A1, CX3CL1, CXCL12, NR2F2) across the pseudo-time. **(O)** Gene dynamic analysis of EC population transition. The left part was a heatmap displaying the expression dynamics of DEGs with distinct patterns along the pseudo-time. The right part was GO and KEGG enrichment terms of each cluster.

To elucidate the dynamics of DPCDI genes, we defined the sub-populations of CM and EC (Supplementary Figure S5; Supplementary Figure S6), representing two major DPCDI-enriched groups in the LV landscape of HF. The Two sub-populations of CM were identified (Figure 6A), including CM1 and CM2. The expressions of three CM-enriched DPCDI genes (IVNS1ABP, JAK2, and OPTN) were depicted in Figure 6B, showing that CM2 expressed higher levels of IVNS1ABP and JAK2. Notably, CM1 expressed increased levels of mitochondrial oxidative stress-related genes (Figure 6C), such as NDUFA4 and C14ORF2; and CM2 demonstrated greater expressions of HF cardiac genes (Figure 6C), such as NPPA and NPPB. Therefore, CM2 may represent an end-stage CM population, suggesting a vibrant activity of apoptosis. We further performed pseudo-time analysis and the trajectory of two CM sub-populations was constructed (Figure 6D). Interestingly, we found that CM1 was expressed at a relatively earlier pseudo-time, with two branches significantly differentiating from nodes across the pseudo-time. Early branch was largely dominated by CM1, whereas the late branch was mainly composed of CM2. Next, we examined the dynamics of cardiac genes and DPCDI genes along the pseudo-time. Considering these cardiac genes, we showed that MYL4, MYL9, NPPA NPPB, and TNNT1 progressively increased (Figure 6E), representing a shift towards CM1 to CM2. As shown in Figure 6F, IVNS1ABP and JAK2 were gradually decreased along the pseudo-time, suggesting their potential in mediating CM differentiation. Additionally, STAT3 expressed relatively lower expression in CM2 and almost absent in CM1. Next, we partitioned genes that were significantly expressed along the pseudo-time trajectory into four different clusters (Figure 6G). During the CM differentiation, genes in cluster 4 (C4), such as IVNS1ABP and JAK2, were progressively decreased from an intermate state to an end state, and significantly enriched in terms related to the cytoskeleton. Additionally, cluster 1 (C1) including HF cardiac genes, were progressively elevated, and mostly enriched for terms related to muscle contraction and cytoskeleton. We also identified two sub-populations of EC (Figure 6H), including EC1 and EC2. As shown in Figures 6I,J, CXCL12 (an EC-enriched DPCID gene) was mostly expressed in the EC2 sub-population. Notably, EC1 exhibited an increased expression of Ca2+ ATPase-related genes (Figure 6K), such as CSRP3, PLN, and ATP2A2. EC1 also highly expressed ANKRD1, an EC activation factor contributing to repressing cardiac gene expression, which promotes cardiac modeling. As depicted in Figure 6K, EC2 was characterized by cell migration-related genes (CX3CL1 and CXCL12) and vessel morphogenesis-related genes (COL4A1 and NR2F2). Pseudo-time analysis was then performed to construct the trajectory of two EC sub-populations (Figure 6L). Notably, the early branch with lower pseudo-time was largely dominated by EC2, whereas the late branch was mainly composed of EC1. We next examined the dynamics of EC1 and EC2 marker genes along the pseudo-time, as depicted in Figures 6M,N. We showed that EC2 marker genes, especially CXCL12, progressively decreased during the state transformation. Next, the differential expressed genes across the pseudo-time were partitioned into four distinct clusters, as shown in Figure 6O. EC2 maker genes were allocated to C2, demonstrating a stronger involvement in ribosome-related functions. EC1 marker genes were clustered to C4 that were progressively elevated, which displayed an enrichment in muscle contraction-related terms.

### 3.6 Genetic predisposition towards CXCL12, JAK2, and STAT3 were found to casually associated with HF

**FIGURE 7:**
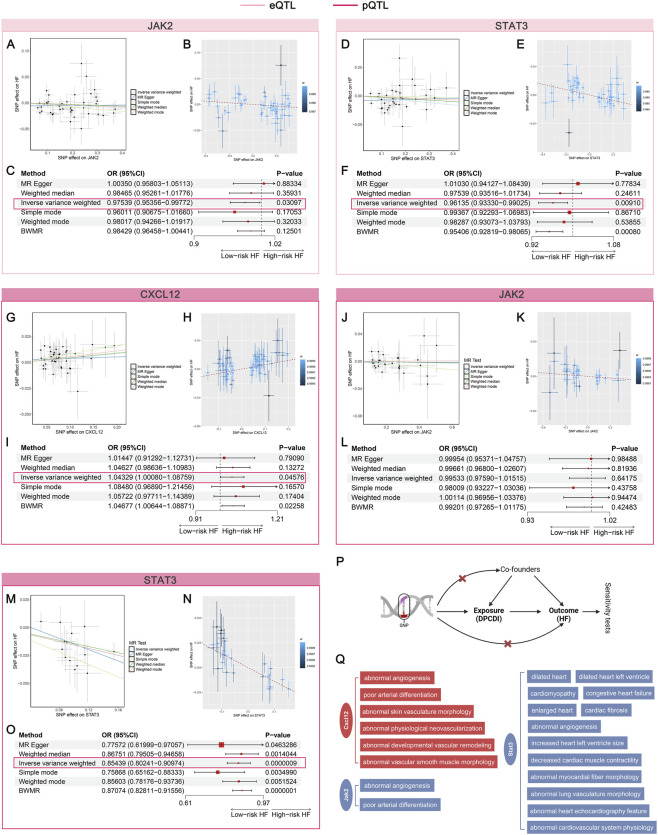
Mendelian randomization assessing the causal relationship between DPCDI genes and HF. **(A–C)** MR analysis of JAK2 (eQTL), in which A and B represent the correlation between the SNP effect of JAK2-eQTL (x-axis) and HF (y-axis) based on 2SMR and BWMR respectively, C was a forest plot summarizing the OR effects and P-values of 2SMR and BWMR on the causal relation-ship between JAK2-eQTL and HF. **(D–F)** MR analysis of STAT3 (eQTL), in which D and E represent the correlation between the SNP effect of STAT3-eQTL (x-axis) and HF (y-axis) based on 2SMR and BWMR respectively, F was a forest plot summarizing the OR effects and P-values of 2SMR and BWMR on the causal relationship between STAT3-eQTL and HF. **(G–I)** MR analysis of CXCL12 (pQTL), in which G and H represent the correlation between the SNP effect of CXCL12-pQTL (x-axis) and HF (y-axis) based on 2SMR and BWMR respectively, I was a forest plot summarizing the OR effects and P-values of 2SMR and BWMR on the causal relationship between CXCL12-pQTL and HF. **(J–L)** MR analysis of JAK2 (pQTL), in which J and K represent the correlation between the SNP effect of JAK2-pQTL (x-axis) and HF (y-axis) based on 2SMR and BWMR respectively, L was a forest plot summarizing the OR effects and P-values of 2SMR and BWMR on the causal relationship between JAK2-pQTL and HF. **(M–O)** MR analysis of STAT3 (pQTL), in which M and N represent the correlation between the SNP effect of STAT3-pQTL (x-axis) and HF (y-axis) based on 2SMR and BWMR respectively, O was a forest plot summarizing the OR effects and P-values of 2SMR and BWMR on the causal relationship between STAT3-pQTL and HF. **(P)** Schematic illustration of this flow. **(Q)** Cardiovascular phenotypes of CXCL12, JAK2, STAT3 knockout from the MGI database. Red and blue stand for a risk and protective factor of HF, respectively.

To investigate the causal relationship between genetically proxied DPCDI expression and HF risk, we systematically applied 2SMR and BWMR. Among the DPCDI genes, the results of CXCL12 (pQTL), JAK2 (eQTL and pQTL), and STAT3 (eQTL and pQTL) were demonstrated to be statistically significant, suggesting high-support evidence for a genetic association linked to HF. Using 2SMR, the effect sizes of the SNP characteristics of eQTL expressions (JAK2, STAT3) and HF risk were negatively related, which were depicted in Figures 7A,D. The forest plot, leave-one-out plot, and funnel plot of SNP characteristics can be found in Supplementary Figure S7 and Supplementary Figure S8. The negative relationship was also demonstrated using the BWMR method (Figures 7B,E). Using the IVW approach, with the highest statistical power, we found that genetic predisposition toward mRNA expressions of JAK2 and STAT3 significantly decreased the risk of HF (Figures 7C,F; OR <1, P < 0.05). Additionally, the direction of effect estimate through IVW was consistent with the other five approaches. Horizontal heterogeneity and pleiotropy were not observed (Supplementary Table S9; Supplementary Table S10), which guarantees the analytic reliability.

With regard to the pQTL data on CXCL12, JAK2, and STAT3, we found that the effect size of the SNP characteristics of CXCL12 was positively associated with HF (Figure 7G), whereas the SNPs of JAK2 (Figure 7J) and STAT3 (Figure 7M) were negatively correlated to HF. The forest plot, leave-one-out plot, and funnel plot of these SNP characteristics can be found in Supplementary Figure S9 (pQTL of CXCL12), Supplementary Figure S10 (pQTL of JAK2), and Supplementary Figure S11 (pQTL of STAT3). Consentient with 2SMR, the relationships of CXCL12, JAK2, and STAT3 were observed in BWMR, which were respectively shown in Figures 7H,K,N. Using the IVW approach, we found that genetic predisposition toward protein expressions of CXCL12 significantly increased the HF risk (Figure 7I; OR > 1, P < 0.05). Though the estimated direction of JAK2-pQTL was consistent with the eQTL data, no statistical significance was shown (Figure 7L; OR < 1, P > 0.05). Interestingly, we showed that genetically proxied higher protein expression of STAT3 also significantly decreased the HF risk (Figure 7O; OR < 1, P < 0.05), which was corroborate with the eQTL data. Also, we did not note heterogeneity and pleiotropy in the analyses of pQTL data on CXCL12, JAK2, and STAT3 (Supplementary Table S11; Supplementary Table S12; Supplementary Table S13). The workflow was shown in Figure 7P. Furthermore, the MGI database was queried to identify the cardiovascular phenotypes relevant to the knock-out of CXCL12, JAK2, and STAT3 genes in mouse models (Figure 7Q). Abnormal angiogenesis and poor arterial differentiation were induced after the knockout of CXCL12 and JAK2. More important traits, such as dilated heart and cardiomyopathy, occurs in STAT3-knockout mice, suggesting its crucial role in HF progression. Altogether, our data indicates that CXCL12 plays a risk factor contributing to HF, but JAK2 and STAT3 act protective factors against HF.

### 3.7 Mouse model validation of Cxcl12, Jak2, and Stat3 expression

**FIGURE 8:**
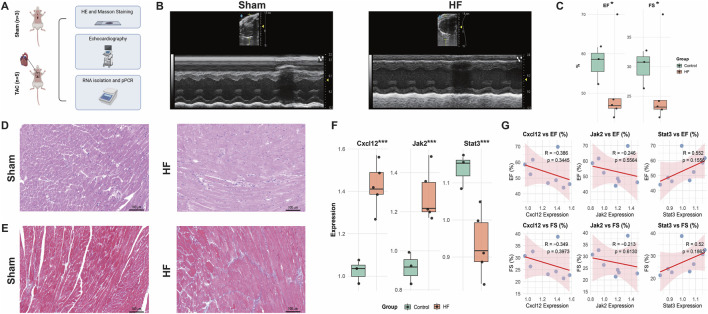
Animal Validation of Cxcl12, Jak2, and Stat3 expressions in the HF context. **(A)** Study flow. **(B,C)** Echocardiography of the sham and HF mouse models. **(D)** HE staining of the sham and HF mouse models. **(E)** Masso staining of the sham and HF mouse models. **(F)** Expressions of Cxcl12, Jak2, and Stat3 in the sham and HF mouse models. **(G)** Correlations between gene expressions and echocardiography indicators. *, *P* < 0.05; **, *P* < 0.01; ***, *P* < 0.001.

To investigate the expression profile of DPCDI-core genes (CXCL12, JAK2, STAT3) in HF, we established TAC-induced HF mouse models. The experimental flow is outlined in Figure 8A. Echocardiography confirmed significantly impaired cardiac function in the TAC group compared to sham-operated controls, as evidenced by reduced ejection fraction (EF) and fractional shortening (FS) (Figures 8B,C; *P* < 0.05). Histological analysis revealed marked cardiomyocyte hypertrophy and inflammatory cell infiltration by H&E staining (Figure 8D), while Masson’s trichrome staining demonstrated extensive interstitial fibrosis (Figure 8E). Furthermore, qPCR analysis of cardiac tissue showed significant upregulation of Jak2 and Cxcl12 mRNA levels and downregulation of Stat3 mRNA levels (Figure 8F; all *P* < 0.001). As depicted in Figure 8G, correlation analysis revealed negative correlations between the expression levels of Jak2 and Cxcl12 and cardiac function parameters (EF and FS), whereas a positive correlation was observed between Stat3 expression and cardiac function (EF and FS). Taken together, these findings suggest that elevated Jak2 and Cxcl12 expression may promote HF progression, whereas Stat3 expression may exert a protective effect against HF.

### 3.8 Therapeutic implications of CXCL12 and STAT3 in mitigating HF

**FIGURE 9:**
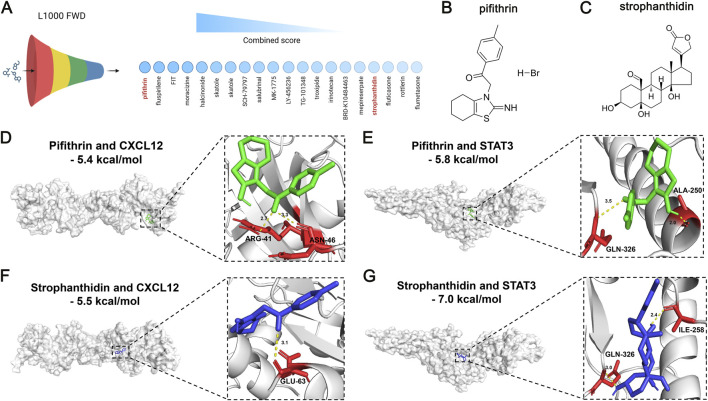
Therapeutic value of DPCDI genes using small-molecule drug prediction and molecular docking. **(A)** The top 20 most significant small-molecule drugs with signature similarity of STAT3 upregulation and CXCL12 downregulation by the L1000fwd database. Pifithrin and strophanthidin were selected. **(B)** Chemical structure of pifithrin. **(C)** Chemical structure of strophanthidin. **(D)** Molecular docking between pifithrin and CXCL12. **(E)** Molecular docking between pifithrin and STAT3. **(F)** Molecular docking between strophanthidin and CXCL12. **(G)** Molecular docking between strophanthidin and STAT3.

Given the risk role of CXCL12 and the protective role of STAT3 against HF, we next sought to explore their therapeutic values for HF treatment. The L1000 FWD database was employed to predict the potential small-molecule drugs with opposite patterns to reverse the upregulation of CXCL12 and downregulation of STAT3 in HF (Figure 9A). The detailed information on the top 20 predicted drugs, including the similarity score, P-value, Q-value, Z-score, and combined score, was summarized in Supplementary Table S14. Considering the toxicity and availability of these drugs, we selected pifithrin and strophanthidin as the candidate drugs. The two-dimensional structures of pifithrin and strophanthidin can be found in Figures 9B,C, respectively. Next, we performed molecular docking to validate the predicted drug-gene interactions. The binding patterns of pifithrin-CXCL12 (Figure 9D, left panel), pifithrin-STAT3 (Figure 9E, left panel), strophanthidin-CXCL12 (Figure 9F, left panel), and strophanthidin-STAT3 (Figure 9G, left panel) were visualized through molecular docking. Importantly, the binding affinities of these dockings were all lower than −5 kcal/mol, indicating reliable interactions. Specifically, pifithrin formed two hydrogen bonds with CXCL12 (Figure 9D, right panel): with ARG-41 and ASN-46 residues at the distance of 2.7 Å and 3.3 Å. Pifithrin formed two hydrogen bonds with STAT3 (Figure 9E, right panel): with ALA-250 and GLN-326 residues at the distance of 2.0 Å and 3.3 Å. Moreover, strophanthidin only formed one hydrogen bond with CXCL12 (Figure 9F, right panel): with GLU-63 residue at a distance of 3.1 Å. Lastly, strophanthidin formed two hydrogen bonds with STAT3 (Figure 9G, right panel): with ILE-258 and GLN-326 residues at the distance of 2.4 Å and 3.0 Å. Thus, pifithrin and strophanthidin may serve as a potential therapeutic candidate for further investigation, repressing the CXCL12 elevation and STAT3 decrease in HF.

## 4 Discussion

HF, a complex clinical syndrome of global epidemic proportions, poses a significant health burden due to its high prevalence and heterogeneous etiology. While management strategies have advanced considerably in recent decades (Bozkurt, 2024), our understanding of its molecular mechanisms remains limited by cardiac complexity (Zhang et al., 2024), hindering precise diagnosis and effective treatment. This underscores the urgent need for novel predictive and therapeutic targets.

Advances in sequencing and bioinformatics have enabled the identification of molecular signatures for disease prediction from omics data (Pilarczyk et al., 2022). Consequently, numerous mRNA signatures derived from transcriptomic assays have been proposed for HF prediction. However, signatures derived from disparate specimen types (such as LV tissues and myocytes isolated from LV (Fan and Hu, 2022)) may lack diagnostic specificity due to cellular heterogeneity during disease progression, limiting their reliability. To address this, we exclusively curated LV-derived gene expression profiles.

Given the established role of programmed cell death (PCD) in cardiovascular disorders (Del Re et al., 2019) and its potential significance in HF, we hypothesized that PCD-related genes could aid in HF identification, stratification, and therapy. We focused on four druggable PCD forms: apoptosis, necrosis, ferroptosis, and autophagy. Ten independent HF cohorts (429 controls, 694 HF cases) from GEO were designated as training, testing, and external validation sets to develop a DPCDI. In the existing studies, the modeling of signature mostly depended on personal preferences (Wang et al., 2022). To avoid this bias, we collected 12 prevalent ML algorithms to construct a predictive signature. Upon packing and benchmarking 12 ML approaches into 113 algorithmic combinations, we successfully designed an integrative computational framework, as our previous studies of building signatures to identifying patients with acute myocardial infarction (Zhu et al., 2024). Ultimately, the optimal combination of Lasso and RF algorithms achieved the highest average AUC score (=0.9815) across the training and testing cohorts, and also performed excellent external validation cohorts. We termed this optimal combination DPCDI, which was fitted on the expressions of 15 DPCD-related genes (CALCOCO2, VPS13D, CLU, STAT3, OPTN, UBB, CXCL12, PPP1R15A, ATF4, IVNS1ABP, HMGB2, JAK2, EXOC7, ENO1, and TPCN1). Interestingly, we found these 15 DPCDI genes were differentially expressed in HF, and largely associated with apoptosis and autophagy. This data underscores the intricate plays between these DPCDI genes in apoptosis and autophagy that contribute to HF.

Overfitting often occurs when establishing predictive signatures, reflected in the high predictive accuracy of the discovery cohort but generalized poor encountering external data (Deo, 2015). Using an integration of Lasso and RF, we minimize the abundant information and identify the most significant genes to generate DPCDI. Benchmarking against 50 published HF signatures revealed DPCDI’s superior generalizability (AUC >0.85 across all cohorts). Notably, while model four model 4 (a 14-aging gene signature by Yu *et al.*) (Yu et al., 2024b) and model 7 (a 17-gene signature by Portokallidou *et al.*) (Portokallidou et al., 2023) ranked highly, their external validation performance was suboptimal, likely due to overfitting. In contrast, our DPCID also presents a good predictive performance regarding the external validation. DPCDI’s robustness stems from Lasso’s L1 regularization (reducing dimensionality, preventing overfitting) (Li et al., 2022) and RF’s handling of unbalanced cohorts (Biau and Scornet, 2016). The combined approach outperformed either algorithm alone (Lasso with AUC = 0.9299; RF with AUC = 0.9599).

The molecular subtype of HF remains incompletely explored. We investigated whether DPCDI contributes to HF molecular subtyping. NMF partitioned 657 HF patients into two divergent subtypes (C1, N = 297; C2, N = 360). DPCDI genes were differentially expressed between subtypes, with two HF markers (NINT2 and EGFR) significantly elevated in C2 versus C1. Given DPCDI’s role in apoptosis and autophagy, we used GSEA and ssGSEA to compare molecular characteristics between DPCDI-derived subtypes. C2 showed greater enrichment in apoptosis, autophagy, mitophagy, and chemokine signaling, but less enrichment in autophagy regulation. Conversely, C1 exhibited the opposite pattern. Studies have showed that apoptosis and autophagy cooperate or antagonize, controlling the cell fate (Noguchi et al., 2020; Nikoletopoulou et al., 2013). Mitophagy, central to mitochondria quality control (Wanderoy et al., 2020), balances apoptosis and autophagy. Based on this evidence, we speculated that the dysregulated mitophagy in C2 exacerbates apoptosis.

Integrating KEGG and literature evidence, we outlined a mechanistic overview of DPCDI gene crosstalk with enriched pathways. HMGB2, IVNS1ABP, ATF4, and PPP1R15A contribute to intrinsic apoptosis. Downregulated ATF4 relieves BCL2L1 inhibition of pro-apoptotic factors BAX and BAK, stimulating mitochondrial outer membrane permeabilization (MOMP) (Yamazaki and Galluzzi, 2022). The upregulation of HMGB2 and PPP1R15A aggravates intrinsic apoptosis through stimulating RELA and BID (Yagi et al., 2003), respectively. INVS1ABP has been found to stabilize actin to inhibit intrinsic apoptosis (Hotter et al., 2020). In C2 patients, the decreasing of INVS1ABP may lose its protective function, and upregulated PPP1R15A may induce apoptosis. Also, we depicted the CXCL12-JAK2-STAT3 axis in the chemokine signaling activates intrinsic apoptosis. Intriguingly, despite activation of CXCL12 and JAK2, STAT3 was inhibited, suggesting a dysphosphorylation of STAT3. STAT3 exerts cytoprotective effects through anti-apoptosis, but its dysregulation induces dilated cardiomyopathy and adverse remodeling post-myocardial infarction (Haghikia et al., 2011; Haghikia et al., 2014). We speculated PTPN2 and SOCS3 inhibit the JAK2-STAT3 activities (Zhang et al., 2018; Carow and Rottenberg, 2014). Regarding C2 patients, we found that CXCL12 and JAK2 increase significantly compared to C1, but STAT3 is downregulated. In this regard, a lower level of STAT3 attenuates its inhibition of apoptosis and thus exacerbates apoptosis.

Additionally, we demonstrated that ENO1, CLU, and UBB, which belong to the SUPER family as apoptotic cell surface markers, were differentially expressed in HF to induce ubiquitination and apoptosis (Díaz-Ramos et al., 2012). Particularly, UBB was significantly elevated in HF, suggesting a higher ubiquitination. VPS13D, a ubiquitin-binding gene in mitochondrial clearance (Anding et al., 2018), was decreased in HF. EXO7 serves as a key component of the mitophagy scaffold (Farré and Subramani, 2011), and was found to be upregulated in HF. Except for the decrease in ENO1, there were no significant changes regarding the mitophagy-upstream regulators (CLU, UBB, VPS13D, and EXO7) in C2 compared to C1. Furthermore, we showed that two cargo receptors for mitophagosome recognition, CALCOCO2 and OPTN (Adriaenssens et al., 2024), were decreased in HF. Despite the activation of ubiquitination to mitophagosome formation, the inhibition of two cargo receptors (CALCOCO2 and OPTN) suggests that the impairment cargo of mitophagosome. TPCN1, which controls Ca^2+^ channels to regulate autophagy, shows an upregulation pattern in HF (Xiong and Zhu, 2016). More importantly, we noted that CALCOCO2 and TPCN1 were significantly decreased in C2. Therefore, we concluded that the recruitment and ubiquitination of mitophagosomes continue in C2 patients. However, the cargo regulation is chaotic because of the impaired expressions of CALCOCO2 and TPCN1. Also, the impeded mitophagy regulation might enhance apoptosis (Noguchi et al., 2020). This may explain the low enrichment of mitophagy but the high enrichment of mitophagy and apoptosis in C2.

We next explored DPCDI expression patterns at single-cell resolution to investigate detailed molecular mechanism. Using scRNA-seq, we plotted the LV cell atlas from four HF patients, including CM, EC, FB, SMC, and MP populations. Among DPCDI genes, JAK2, IVNS1ABP, and OPTN showed higher expression in CM, while CXCL12 was enriched in EC and SMC. JAK2 was highly expressed in CM, but its upstream CXCL12 and downstream STAT3 were not correspondingly enriched, prompting investigation into the CXCL12-JAK2-STAT3 axis across populations. JAK-STAT signaling contributes to HF progression (Pang et al., 2023), but cell-to-cell mechanisms are poorly explained. Using CellChat, we analyzed cell interactions. CXCL12-expressing ECs and SMCs bind CXCR4 receptors on MPs, leading to CXCL signaling that promotes MP pro-inflammation (Kim et al., 2014). Pro-inflammatory MPs exhibit NAMPT activity and bind INSR ligands on CMs. INSR activation promotes JAK2-STAT3 signaling (Salminen et al., 2021). We propose that CXCL12-expressing ECs/SMCs stimulate MP pro-inflammation via CXCL12-CXCR4; pro-inflammatory MPs then interact with CMs via NAMPT-INSR to activate JAK2-STAT3 signaling. Pseudo-time analysis inferred trajectories for CM and EC populations. Two CM-expressed DPCDI genes (IVNS1ABP and JAK2) decreased dynamically during CM transition from oxidative stress to failure. CXCL12, an EC-expressed DPCDI gene, declined during EC transformation from migration and vessel morphogenesis. This indicates roles for IVNS1ABP and JAK2 in CM differentiation, and CXCL12 in EC differentiation.

To assess DPCDI druggability, we performed two large population-based MR studies at mRNA and protein levels. Using eQTL datasets, we identified negative causal associations between JAK2/STAT3 mRNA expression and HF risk. pQTL datasets validated protective roles of JAK2/STAT3 proteins against HF. CXCL12 protein level positively correlated with HF prevalence. Knockout of CXCL12, JAK2, and STAT3 in mice caused cardiovascular phenotypes like abnormal angiogenesis, poor arterial differentiation, and dilated heart, underscoring their pivotal roles in cardiovascular development and validating our MR results. CXCL12 level associates with increased MACEs in CAD patients (Zhang et al., 2022), indicating high risk. HF patients show JAK2 activation but severe STAT3 reduction (Cambi et al., 2012). STAT3 downregulation relates to end-stage HF (Hilfiker-Kleiner et al., 2005; Harhous et al., 2019), highlighting its protective role. Our findings support CXCL12 as a high-risk factor and JAK2/STAT3 as protective factors, suggesting therapeutic targets.

We predicted 20 small-molecule drugs reversing CXCL12 upregulation and STAT3 downregulation in HF. Pifithrin and strophanthidin were promising candidates, showing close affinities with CXCL12 and STAT3 through molecular docking. Pifithrin, a p53 inhibitor, protects against doxorubicin-induced apoptosis and attenuates myocardial ultrastructural alterations (Liu et al., 2004). Strophanthidin, a Na+/K+-ATPase inhibitor, exerts positive inotropic effects on failing human myocardium concentration-dependently (von Lewinski et al., 2007) and enhances anti-tumor activity via STAT3 in HepG2 cells (Reddy et al., 2019). Although predicted and validated *in silico* to interact with CXCL12 and STAT3, their cardio-protective mechanisms through reversing CXCL12/STAT3 expression remain unclear. Detailed investigations into pifithrin and strophanthidin effects on CXCL12 inhibition and STAT3 activation for HF alleviation are needed.

Our study has limitations. First, cohorts were retrospective. Further DPCDI investigation requires larger, multi-ethnic cohorts and clinical correlation. Second, DPCDI gene crosstalk in HF is incompletely explored, and more functional experiments are needed. Third, the therapeutic significance of CXCL12 and STAT3, particularly interactions with pifithrin and strophanthidin, requires detailed pharmacological study.

## 5 Conclusion

In summary, an optimal combination of Lasso and RF that contribute to a 15-gene signature (CALCOCO2, VPS13D, CLU, STAT3, OPTN, UBB, CXCL12, PPP1R15A, ATF4, IVNS1ABP, HMGB2, JAK2, EXOC7, ENO1, and TPCN1) was developed and validated to accurately predict HF, termed as DPCDI. We next leveraged DPCDI to partition HF into two subtypes, in which C2 presents a higher degree of apoptosis and mitophagy, while C1 shows the opposite. At the single-cell resolution, we found the dynamic expression of JAK2 and OPTN among the CM transition to failure status; and CXCL12 in EC transition to vessel morphogenesis. Moreover, we used the MR analysis on the large-scale eQTL and pQTL to elaborate on the causality between DPCDI and HF, with JAK2 and STAT3 as protective factors and CXCL12 as a high-risk factor. Using molecular docking, two agents including pifithrin and strophanthidin were predicted to closely interact with CXCL12 and STAT3. Repressing CXCL12 and stimulating STAT3 through the medication of pifithrin or strophanthidin may represent new therapeutic strategies for HF. Collectively, DPCDI provides a translatable framework for HF diagnosis, molecular subtyping, and precision therapeutics.

## Data Availability

The GEO database (http://www.ncbi.nlm.nih.gov/geo) contains datasets used in this study, including GSE141910, GSE116250, GSE135055, GSE16499, GSE5406, GSE57338, GSE79962, GSE42955, GSE52601, GSE21610, and GSE121893. To ensure productivity of the integrative ML framework and our DPCDI, the source code, scripts, processed datasets, and instructions have been archived in the Zenodo repository (https://doi.org/10.5281/zenodo.17918314).
