# Supplementary material for: DPCDI: an artificial intelligent-derived indicator interpreting the diagnostic, stratification, and therapeutic implications of druggability programmed cell death in heart failure
Source: Front Genet. 2026 Jan 15;16:1753636. doi: 10.3389/fgene.2025.1753636 (PMC12851539; doi:10.3389/fgene.2025.1753636)
Supplement: Supplementary file 2 [file DataSheet1.docx]

Supplementary Material

## Supplementary Tables

Please see external excel file.

## Supplementary Figures


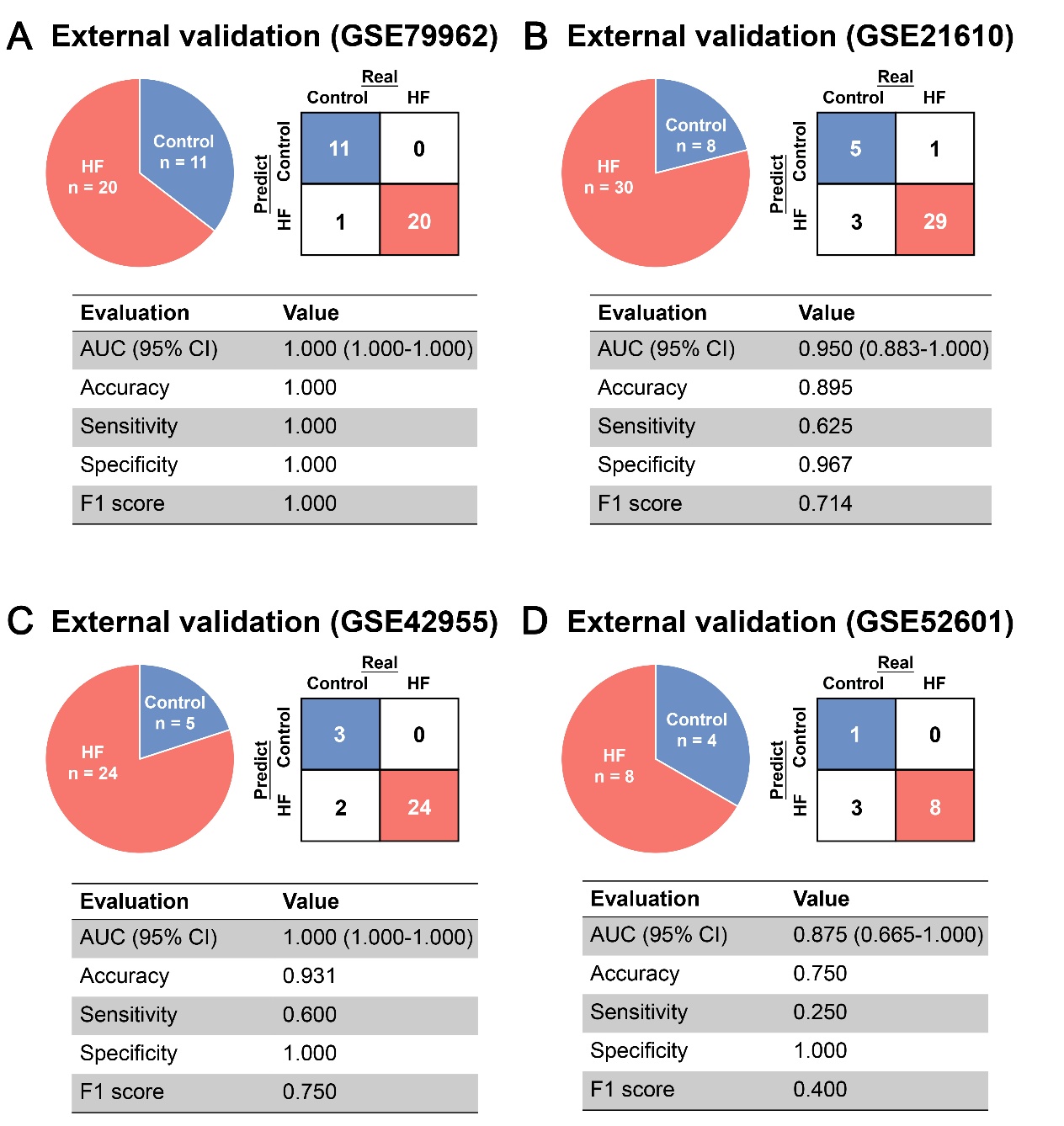


**Supplementary Figure 1.** External validation on DPCDI and clinical characteristics of DPCDI genes. (A) Confusion matrix and diagnostic assessment of DPCDI in the GSE79962 cohort. (B) Confusion matrix and diagnostic assessment of DPCDI in the cohort. (C) Confusion matrix and diagnostic assessment of DPCDI in the cohort. (D) Confusion matrix and diagnostic assessment of DPCDI in the cohort.


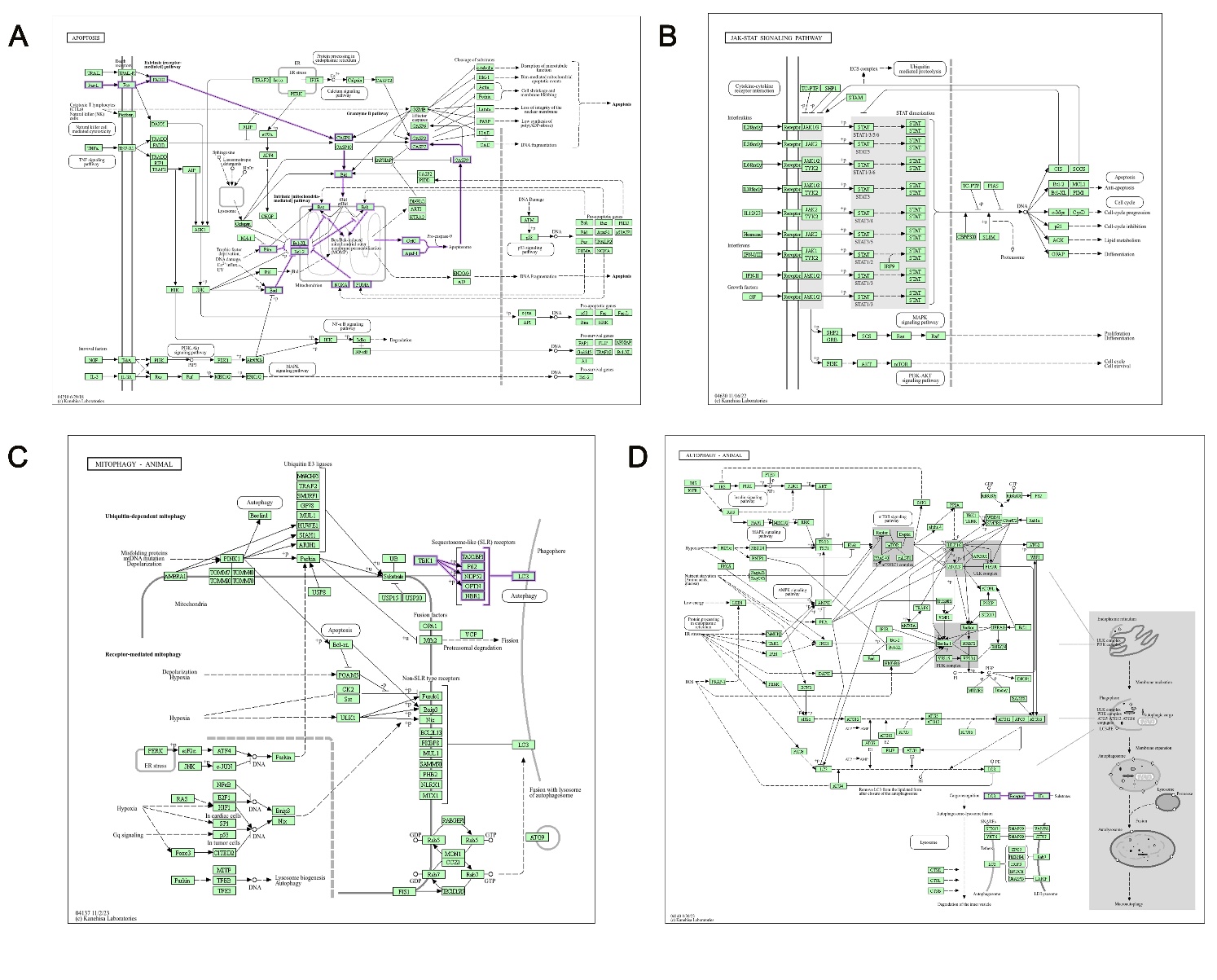


**Supplementary Figure 2. KEGG pathways.** (A) Apoptosis. (B) JAK-STAT signaling pathway. (C) Mitophagy. (D) Autophagy.


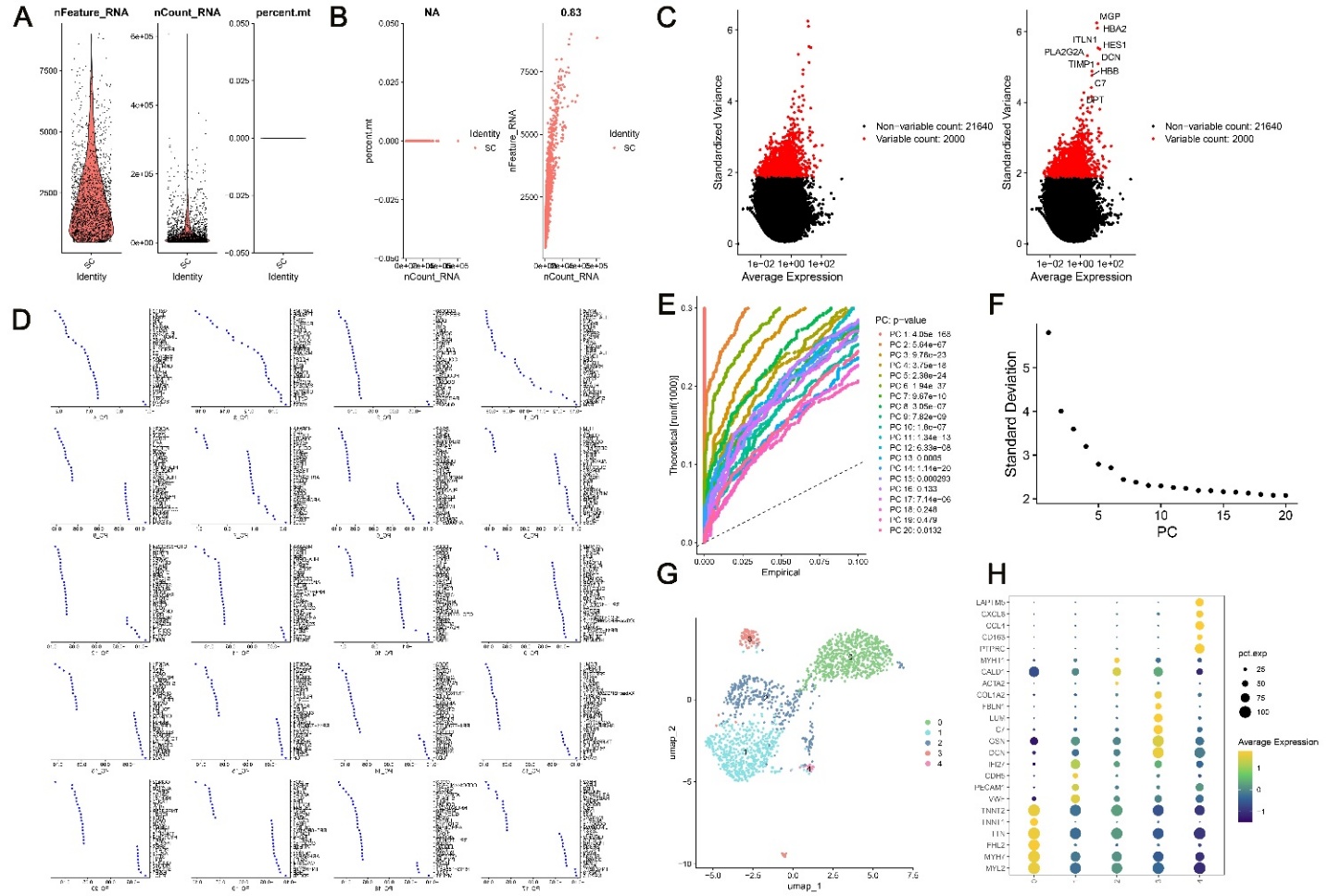


**Supplementary Figure 3.** Quality control, dimensional reduction, and UMAP clustering on LV samples from GSE121893 cohort. (A) Filtering out cells with less than 200 or larger than 5000 feature counts and mitochondrial proportion larger than 20%. (B) Correlation between gene counts and feature counts. (C) Identification of highly variable genes. The red dots represent the top 2000 highly variable genes and the top 10 labeled with gene symbols. (D) The 20 principal components (PCs) on scaling data. (E)Statistical significance plot of PCs indicated the top 15 PCs were determined for clustering (P < 0.05). (F) Elbow plot ranking PCs on the percentage of variance indicated the top 15 PCs were determined for clustering. (G) UMAP clustering based on the top 15 PCs. (H) Annotation of cell clusters.


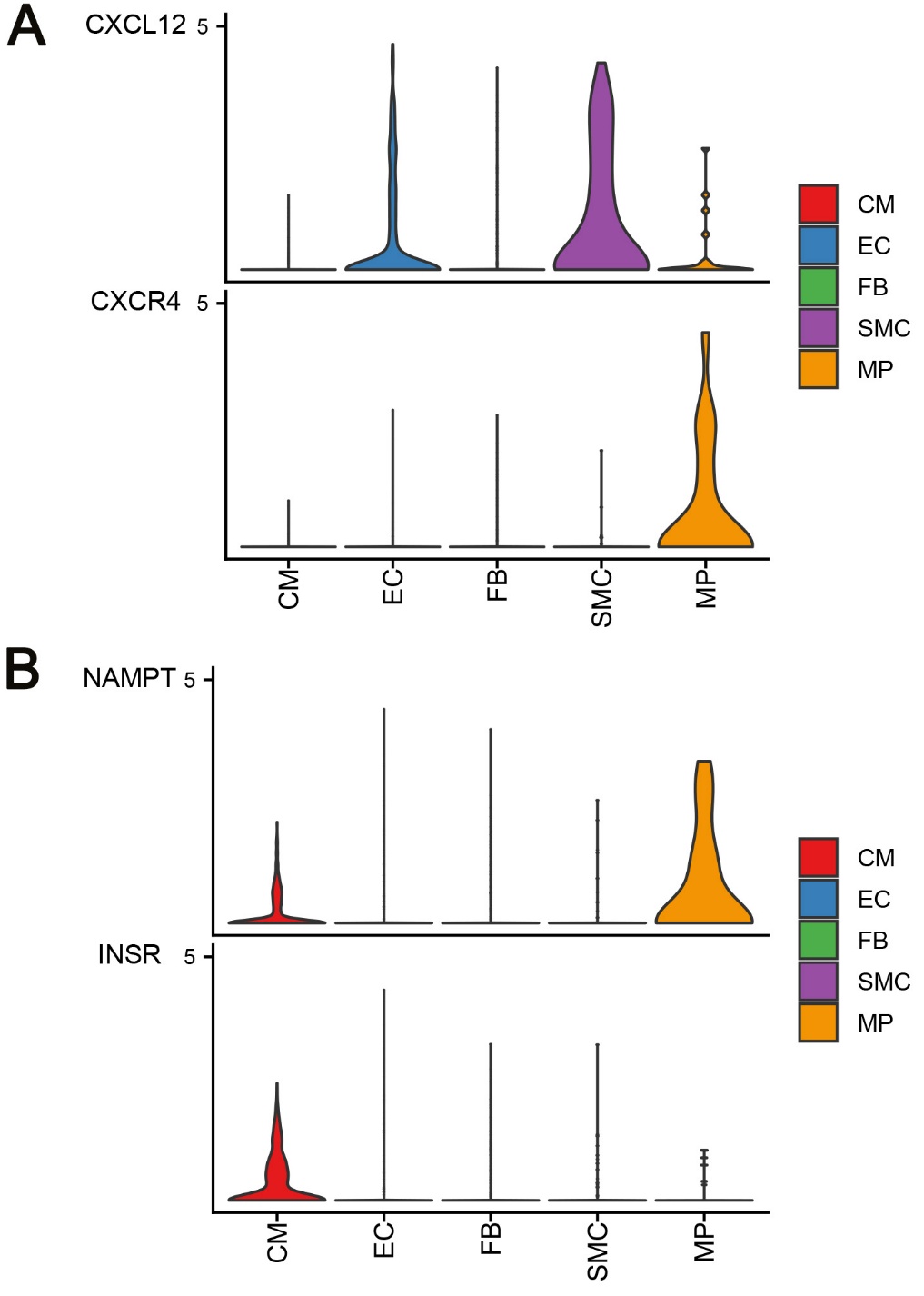


**Supplementary Figure 4.** Expression distributions of ligands and receptors in CXCL and VISFATIN signaling pathways. (A) Expression distributions of CXCL12 ligand and CXCR4 receptor of CXCL signaling pathway. (B) Expression distributions of NAMPT ligand and INSR receptor of VISFATIN signaling pathway.


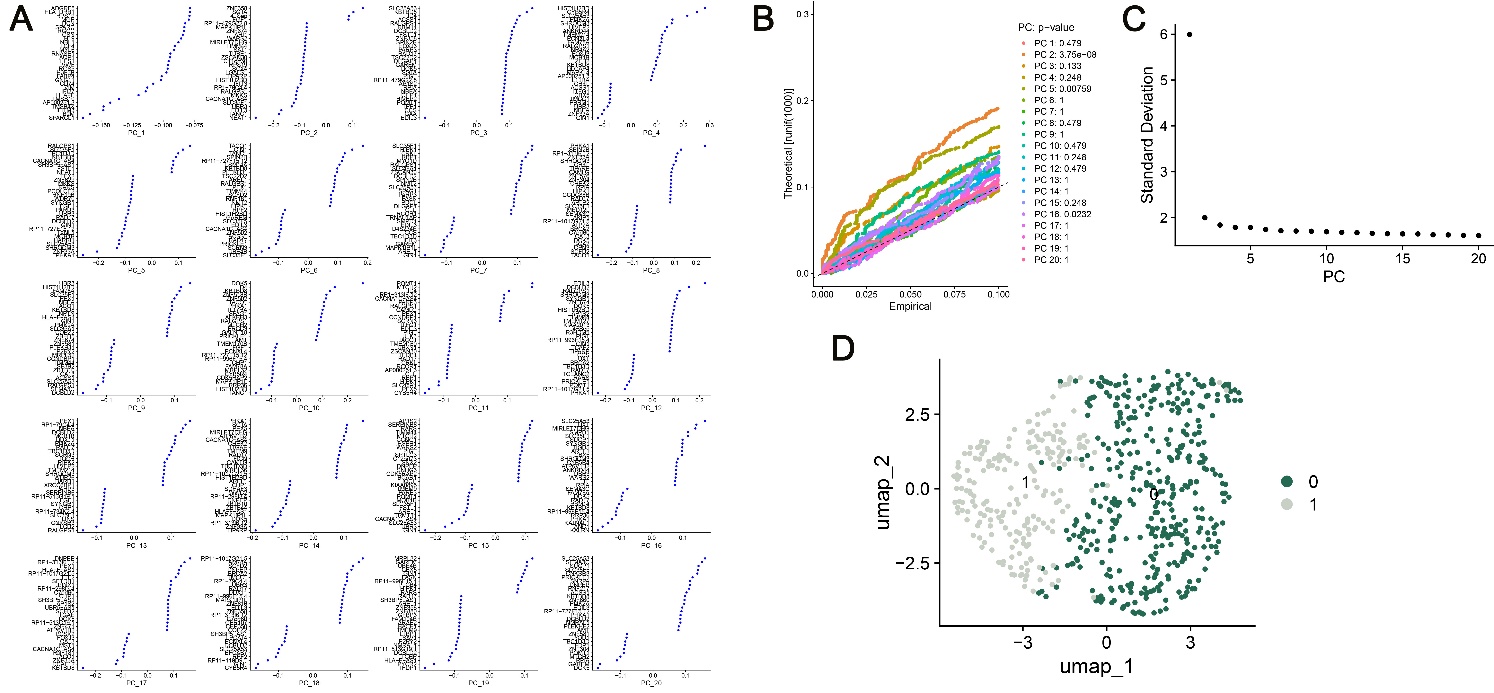


**Supplementary Figure 5.** Dimensional reduction and UMAP clustering on CM population. (A) The 20 principal components (PCs). (B) Statistical significance plot of PCs indicated the top 5 PCs were determined for clustering. (C) Elbow plot ranking PCs on the percentage of variance indicated the top 5 PCs were determined for clustering. (D) UMAP clustering based on the top 5 PCs.


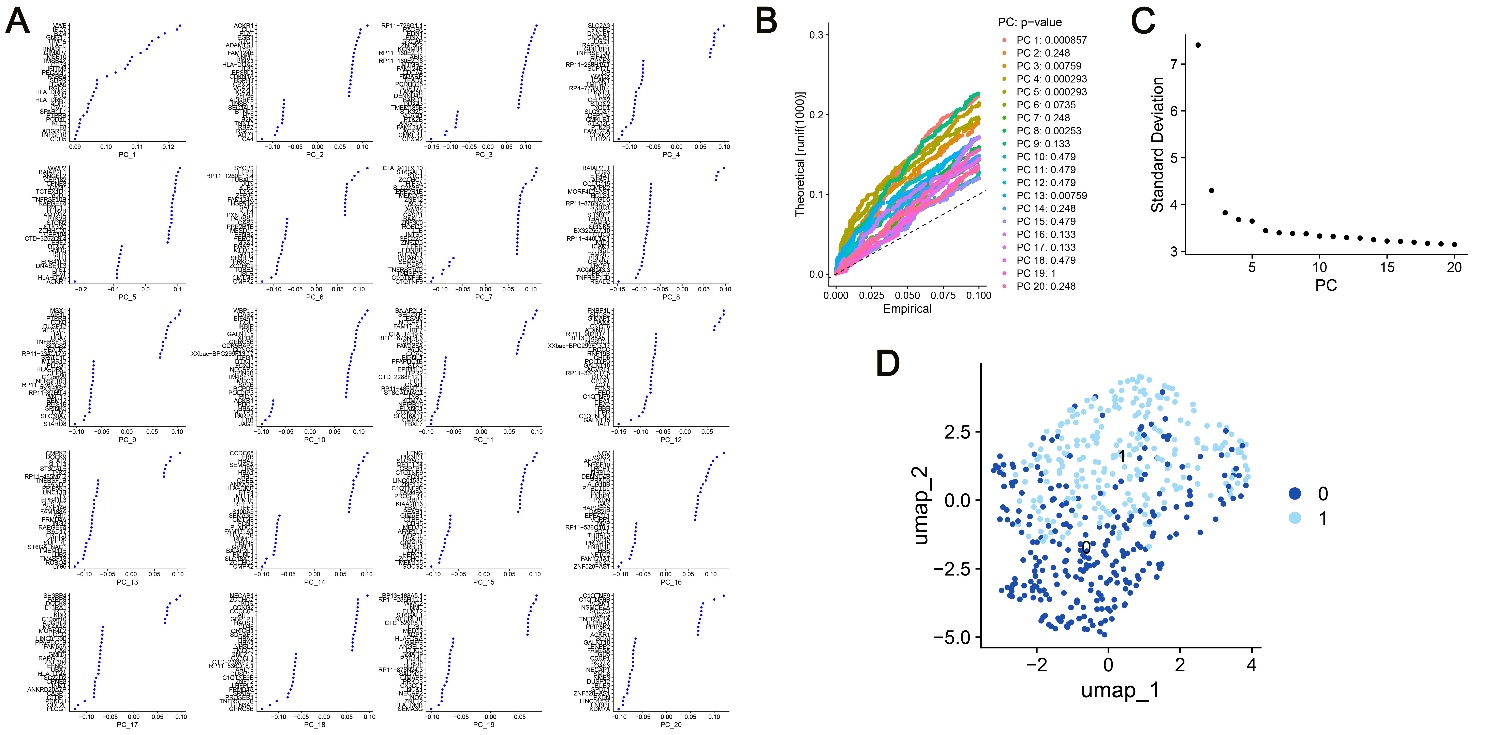


**Supplementary Figure 6.** Dimensional reduction and UMAP clustering on EC population. (A) The 20 principal components (PCs). (B) Statistical significance plot of PCs indicated the top 5 PCs were determined for clustering. (C) Elbow plot ranking PCs on the percentage of variance indicated the top 5 PCs were determined for clustering. (D) UMAP clustering based on the top 5 PCs.


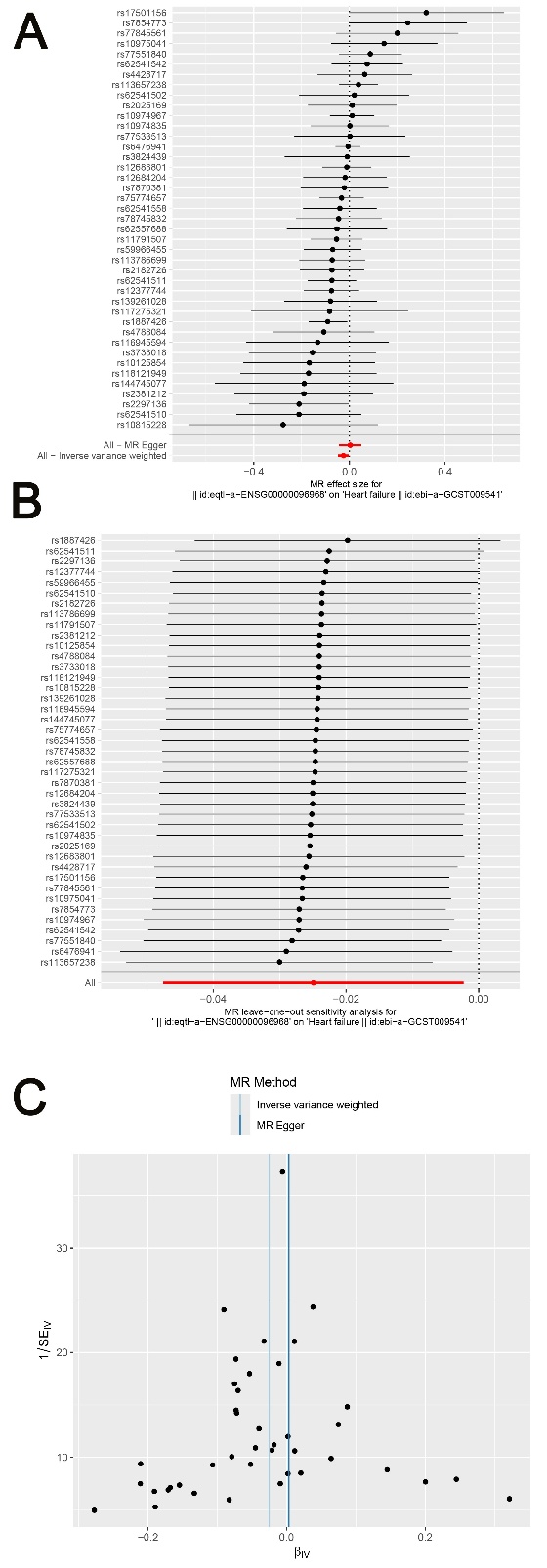


**Supplementary Figure 7.** Forest plot, leave-one-out plot, and funnel plot of SNPs used in 2SMR for JAK2 (eQTL). (A) Forest plot. (B) Leave-one-out plot. (C) Funnel plot.


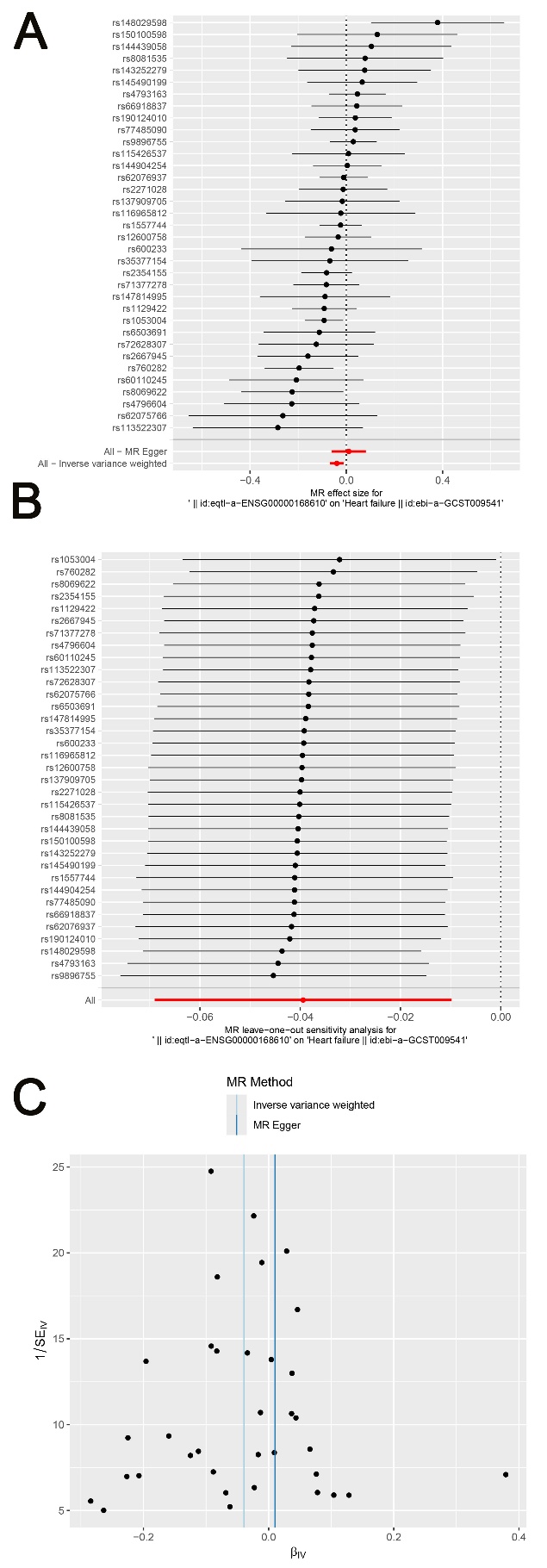


**Supplementary Figure 8.** Forest plot, leave-one-out plot, and funnel plot of SNPs used in 2SMR for STAT3 (eQTL). (A) Forest plot. (B) Leave-one-out plot. (C) Funnel plot.


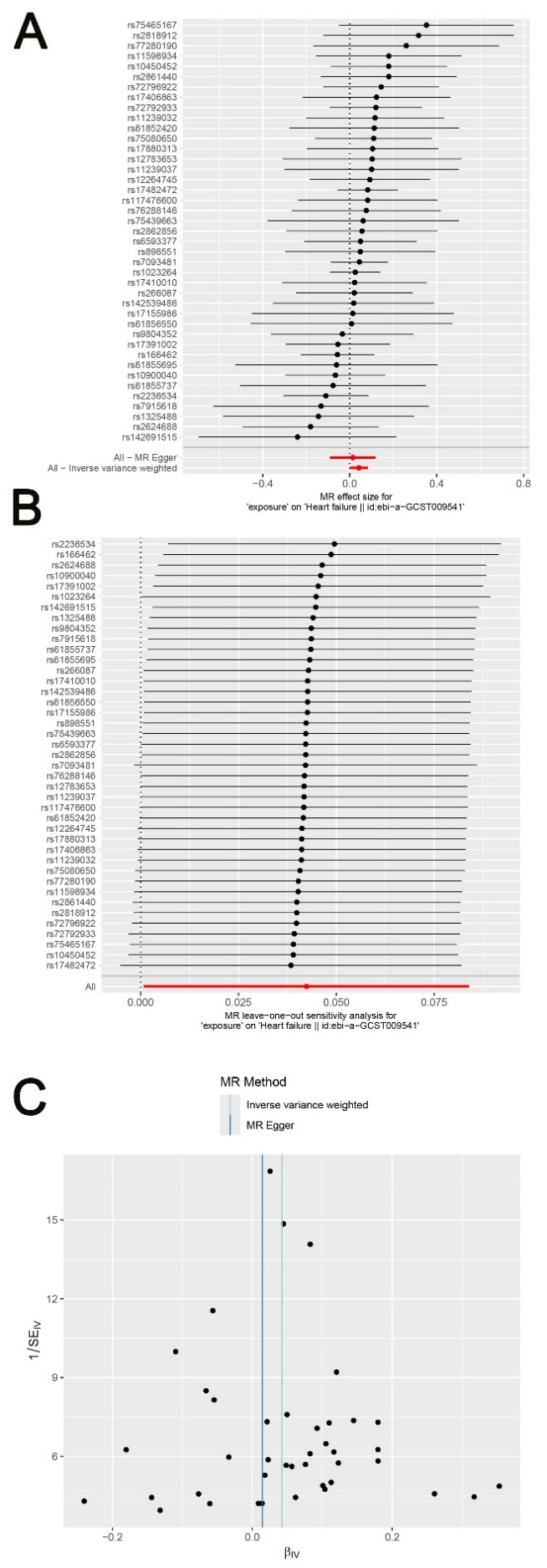


**Supplementary Figure 9.** Forest plot, leave-one-out plot, and funnel plot of SNPs used in 2SMR for CXCL12 (pQTL). (A) Forest plot. (B) Leave-one-out plot. (C) Funnel plot.


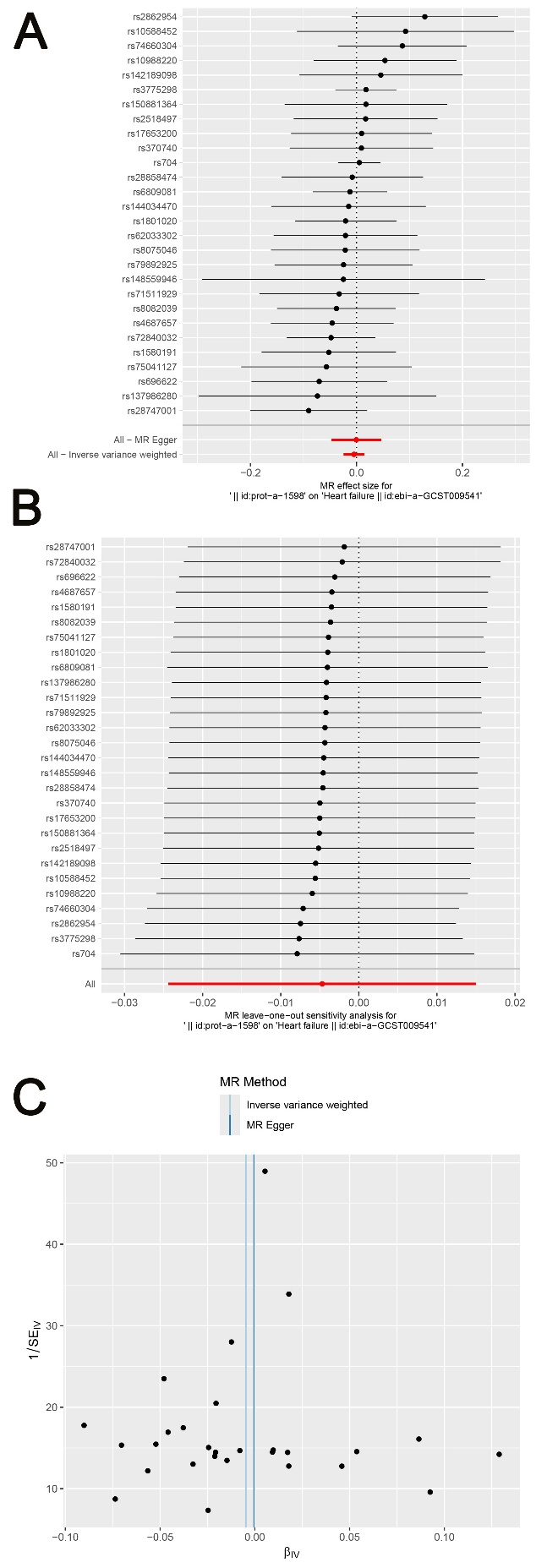


**Supplementary Figure 10.** Forest plot, leave-one-out plot, and funnel plot of SNPs used in 2SMR for JAK2 (pQTL). (A) Forest plot. (B) Leave-one-out plot. (C) Funnel plot.


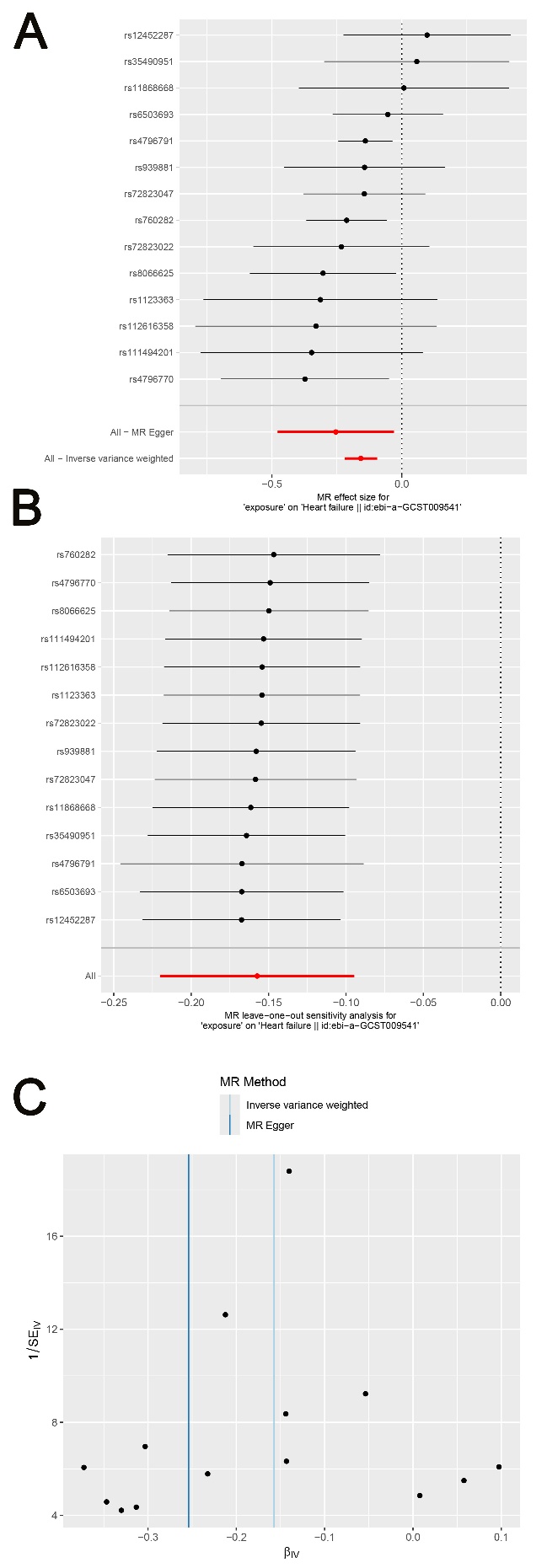


**Supplementary Figure 11.** Forest plot, leave-one-out plot, and funnel plot of SNPs used in 2SMR for STAT3 (pQTL). (A) Forest plot. (B) Leave-one-out plot. (C) Funnel plot.
